# Comparison of the Human Plasma Peptides from the Fit of Fragmentation Spectra versus Accurate Monoisotopic Precursor Mass

**DOI:** 10.1021/acsomega.4c06211

**Published:** 2025-03-10

**Authors:** Zhuo Zhen Chen, Jaimie Dufresne, Peter Bowden, John G. Marshall

**Affiliations:** Research Analytical Biochemistry Laboratory, Department of Chemistry and Biology, Faculty of Science, Toronto Metropolitan University, 350 Victoria Street, Toronto, Ontario M5B 2K3, Canada

## Abstract

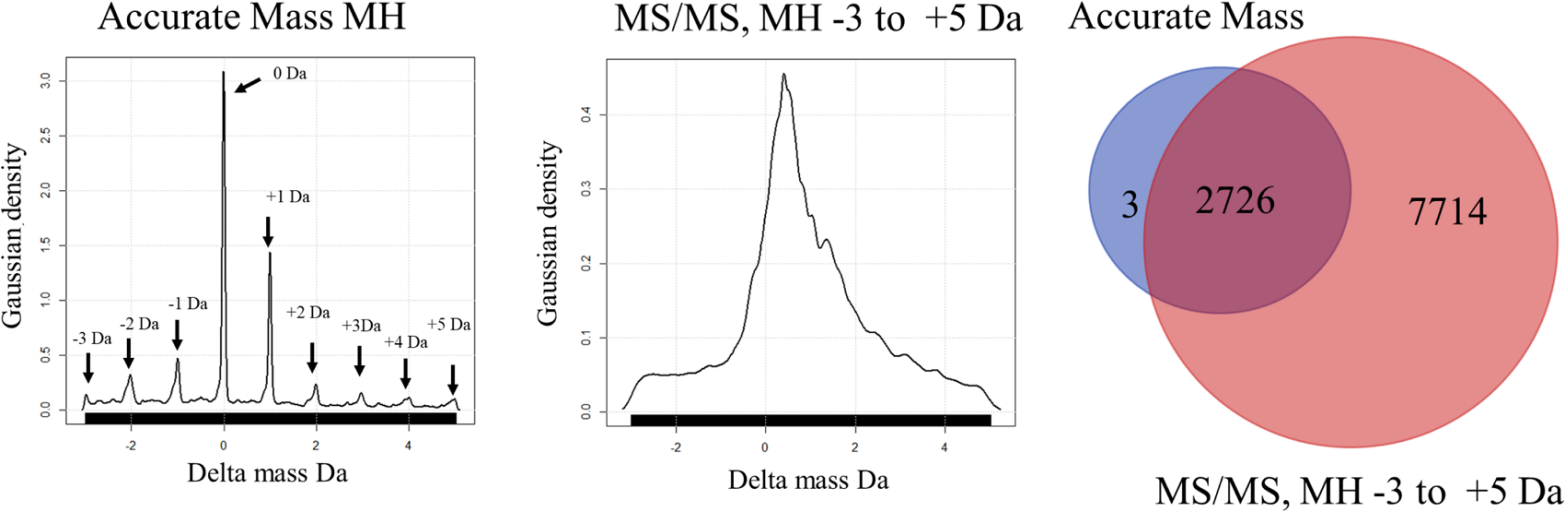

In nature, ionized peptides with heavy isotopes and hydrogen rearrangements show a broad mass distribution with signals at discrete delta mass values from −3 to +5 Da by mass spectrometry (MS). For many peptides, the intensity of the +1 or +2 Da isotope exceeds the signal from the monoisotopic mass. Therefore, there is a need for a method that improves peptide identification from heavy isotopes or hydrogen rearrangements based on the fit of tandem mass spectra. Peptides may be identified using an accurate monoisotopic precursor mass with ≤0.1 Da. However, many peptides with heavy isotopes and H-loss can be identified and enumerated based on the fit of their MS/MS spectra alone in the absence of an accurate precursor monoisotopic mass (i.e., ± 3 Da) using the X!TANDEM MS/MS fitting algorithm. In this study, human plasma samples were analyzed with a highly resolving axially harmonic orbital ion trap (OIT) and a sensitive linear quadrupole ion trap (LIT). The MS/MS fragmentation spectra from the OIT can be fit to peptides from the monoisotopic (±0.1 Da) as well as all other precursor masses with a wide mass tolerance (±3 Da). The resulting delta mass distribution can then be plotted and compared to the predicted distribution of heavy isotopes and hydrogen rearrangements to provide a direct biophysical prediction and test the validity of the fit determined by accepting the best-fit MS/MS spectra. The OIT instrument, which has greater resolution, was sampled at 30 nL per minute, while the more sensitive LIT was sampled at 200 nL per minute. The MS/MS spectra generated by each instrument were fit to peptides within a wide window (±3 Da) using the rigorous X!TANDEM algorithm. The OIT and LIT results were compared in an SQL Server database and corrected against analytical and statistical controls. The delta mass distribution of the peptides with hydrogen rearrangements and heavy isotopes was determined from the fit MS/MS spectra using the R statistical program. The OIT sampled MS and MS/MS spectra from the high-intensity precursor ions by focusing on E7 to E9 detector counts. In contrast, the LIT sampled a range of precursor ion intensities focused from E4 to E7 and thus reached lower ion intensity values. As expected, the precursor mass [M + H]^+^ obtained by the OIT exhibited sharp delta mass peaks at −3, −2, −1, 0, +1, +2, +3, +4, and +5 Da due to naturally occurring heavy isotopes and hydrogen rearrangements. The collection of peptides and proteins identified by OIT and LIT was in qualitative and quantitative agreement with one another, with 99.9% overlap on 2726 protein gene symbols from human plasma and a highly significant relationship by regression analysis. The protein *p*-values, false discovery rate *q*-values, and comparisons to the noise MS/MS analytical control and random MS/MS statistical control confirmed the high-confidence MS/MS identifications from both instruments. MS/MS fragmentation spectra from the OIT were fit to peptides. The resulting precursor ion delta mass distribution showed a precise match to the predicted isotope distributions and hydrogen rearrangements of natural peptides. Thus, analysis of delta mass plots provided powerful biophysical evidence for the accuracy of plasma peptide identification from the fit of the MS/MS spectra alone. The high level of agreement on proteins and peptides and the proportional enumeration between proteins identified by the OIT and those identified independently using a LIT confirmed that plasma peptides and proteins may be identified and quantified from MS/MS spectra alone without the need for an accurate measure of the precursor mass. The greater sensitivity and low cost of searching MS/MS spectra in the absence of an accurate mass mean that it is possible to identify and quantify more proteins for the discovery of proteins in clinical populations.

## Introduction

Natural peptides contain many heavy isotopes^1^ and may undergo hydrogen rearrangements in the gas phase.^2,3^ Thus, only a fraction of peptide ions can be detected by MS/MS based on their monoisotopic mass. Clearly, limiting the analysis to the monoisotopic mass alone may reduce the overall sensitivity of the LC-ESI-MS/MS method for challenging applications like analysis of plasma.^2,3^ The precursor masses of peptides derived from natural sources, for example, human plasma proteins, may have a wide distribution and differ from the monoisotopic mass by many Daltons (e.g., −3 to +5 Da).^2,3^ Analyses of highly purified viral coat proteins,^4^ protein standards,^5,6^ and synthetic peptide standards^7^ provided strong experimental support for the validity of peptide identification from the fit of tandem mass spectra (MS/MS) alone in the absence of an accurate estimate of the precursor peptide mass. Experiments based on results from random protein libraries^8^ that include wide search parameters^9^ and random or “noise” MS/MS spectra^5,6^ provided strong statistical support for the reliable identification of peptides from fit MS/MS spectra alone^10−12^ where confidence builds with replication.^13^ Similarly, statistical methods, for example, the X!TANDEM goodness-of-fit algorithm, provide protein *p*-values that are direct estimates of the type I error rate from the fit of MS/MS spectra and peptides^10^ that may be corrected to false discovery rate (FDR) *q*-value using the well-established method of Benjamini and Hochberg.^14^ The FDR q-value agreed with the results of the Monte Carlo simulation^15−18^ which revealed that false positives were randomly distributed across all peptides in the database while true positive results were sharply focused on a subset of peptides with high observation frequencies.^5,6,8^ The MS/MS spectra from natural peptides, including those with heavy isotopes, were reliably identified without accurate peptide mass measurements^19−21^ but did require correction against the “noise” MS/MS analytical and random MS/MS spectra statistical controls to correct the observation frequency of peptides from large proteins or extensive protein families, e.g., titan (TTN), obscurin (OBSC), microtubule-actin cross-linking factor 1 (MACF1), immunoglobulin (Ig), major histocompatibility complex (MHC) and others.^5,6,22,23^ Taken together, it was possible to analyze the MS/MS spectra alone to identify and enumerate many peptides from most proteins.

There is limited direct biophysical evidence indicating that the fit of peptides from MS/MS spectra alone has a low error rate. Powerful and important findings might be drawn from the delta mass distribution obtained from the fit of MS/MS spectra from the OIT to the known peptides with heavy isotopes and hydrogen rearrangements. If the peptides identified by the trihybrid orbital ion trap (OIT) from the fit of MS/MS spectra alone showed the expected delta mass distributions from heavy isotopes and hydrogen rearrangements, these observations would provide strong biophysical support for identification from peptide fragmentation (MS/MS) without an accurate mass. One strategy that might be used to support the validity of peptides identified from MS/MS spectra with an ion trap might be to compare peptides identified from an analysis of the precursor at the monoisotopic mass versus those with delta mass values consistent with heavy isotopes and hydrogen rearrangements.^2,3^ In addition, the fit of MS/MS spectra with the OIT might be compared to MS/MS spectra using a linear quadrupole ion trap (LIT) that showed excellent agreement but that the LIT was more sensitive.^24^ Similarly the LIT provided comparable results but was more sensitive for targeted proteomics.^25^ Comparisons of low-resolution ion traps to high-resolution quadrupole-time-of-flight (Qq-TOF) or Fourier-transform ion cyclotron resonance (FTICR) revealed that results from these instruments were in agreement on high abundance proteins but that the simple ion trap was more sensitive.^26−29^ The use of the LIT resulted in high observation frequencies for plasma proteins that were also detected by the OIT.^23^ In this study, the direct biophysical measurement of delta mass distribution, the agreement of the monoisotopic MS/MS spectra with those of hydrogen rearrangements and heavy isotopes, the concordance of results from the OIT and LIT, the independent statistical analysis of the FDR *q*-value, and the comparisons to random MS/MS spectra collectively provide an unambiguous demonstration that the peptides and proteins from human plasma may be identified at a low error rate from the fit of MS/MS spectra alone using the OIT or LIT instrument without the need for an accurate measure of monoisotopic mass.

## Results

**Figure 1:**
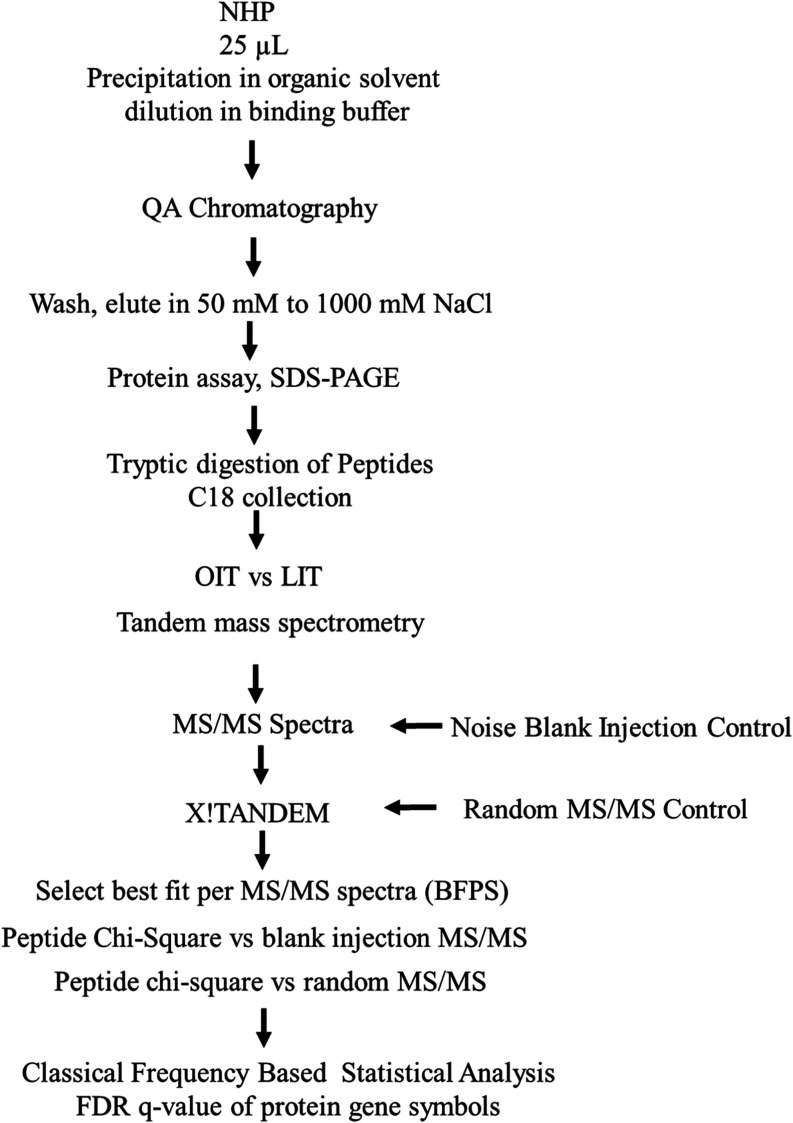
Flowchart of the experiment design to compare the results of OIT versus LIT for the analysis of tryptic peptides from human plasma proteins. Normal human plasma (NHP) was precipitated in ACN and resuspended in 20 mM tris pH 8.85 for protein assays with the addition of urea and ACN prior to digestion. Disposable C18 columns were used to collect peptides for LC-ESI-MS/MS.

The tryptic peptides from human plasma were analyzed by LC-ESI-MS/MS using the accurate mass of the trihybrid OIT and the MS/MS of the OIT and simple LIT; the results were collected and rendered nonredundant in an SQL Server database for comparison. The MS/MS spectra from the two different instruments and the results obtained from the LC-ESI-MS/MS experimental setups were compared using the X!TANDEM algorithm. The Best Fit Per MS/MS Spectra (BFPS) peptides were selected, and the results were then graphically and statistically analyzed using the R statistical system. The steps to making a highly sensitive analysis of human plasma have been examined (Figure 1): The simple ion trap may identity fully tryptic and nontryptic peptides of blood;^27,30^ the methods to precipitate plasma proteins were compared;^31,32^ partition chromatography^27,33^ performed better than depletion chromatography;^34^ the optimal QA chromatography was compared under different salt elution regimes;^22^ the rigorous X!TANDEM algorithm was validated with noise and random spectra using *p*-values versus decoy library;^35,36^ purified peptide and proteins standards were used to select the optimal search parameters of ±3 Da precursor and ±0.5 Da fragments to identify and quantify proteins from MS/MS spectra;^5,6^ optimized tryptic digestions conditions with trypsin alone were established to avoid the digestion of albumin.^27^ The SQR Server removed redundant protein assignments selecting the single best fit of MS/MS spectra^37,38^ and the R Statistical system identified some 13,000 proteins from plasma with the rigorous X!TANDEM algorithm after Chi Square analysis against noise and random MS/MS before computing the gene symbol *p*-value and FDR *q*-value.^39^ The delta mass distribution of peptides identified by the OIT instrument using MS/MS spectra alone exactly matched the predicted distribution of peptides with hydrogen rearrangements and heavy isotopes. The findings generated using the OIT, which has a much higher mass resolution, were in excellent agreement (99.9%) with those from the sensitive LIT where the two instruments identified similar peptides from an overlapping set of human plasma proteins.

### Orbital Ion Trap (OIT) Delta Mass Distribution

**Figure 2:**
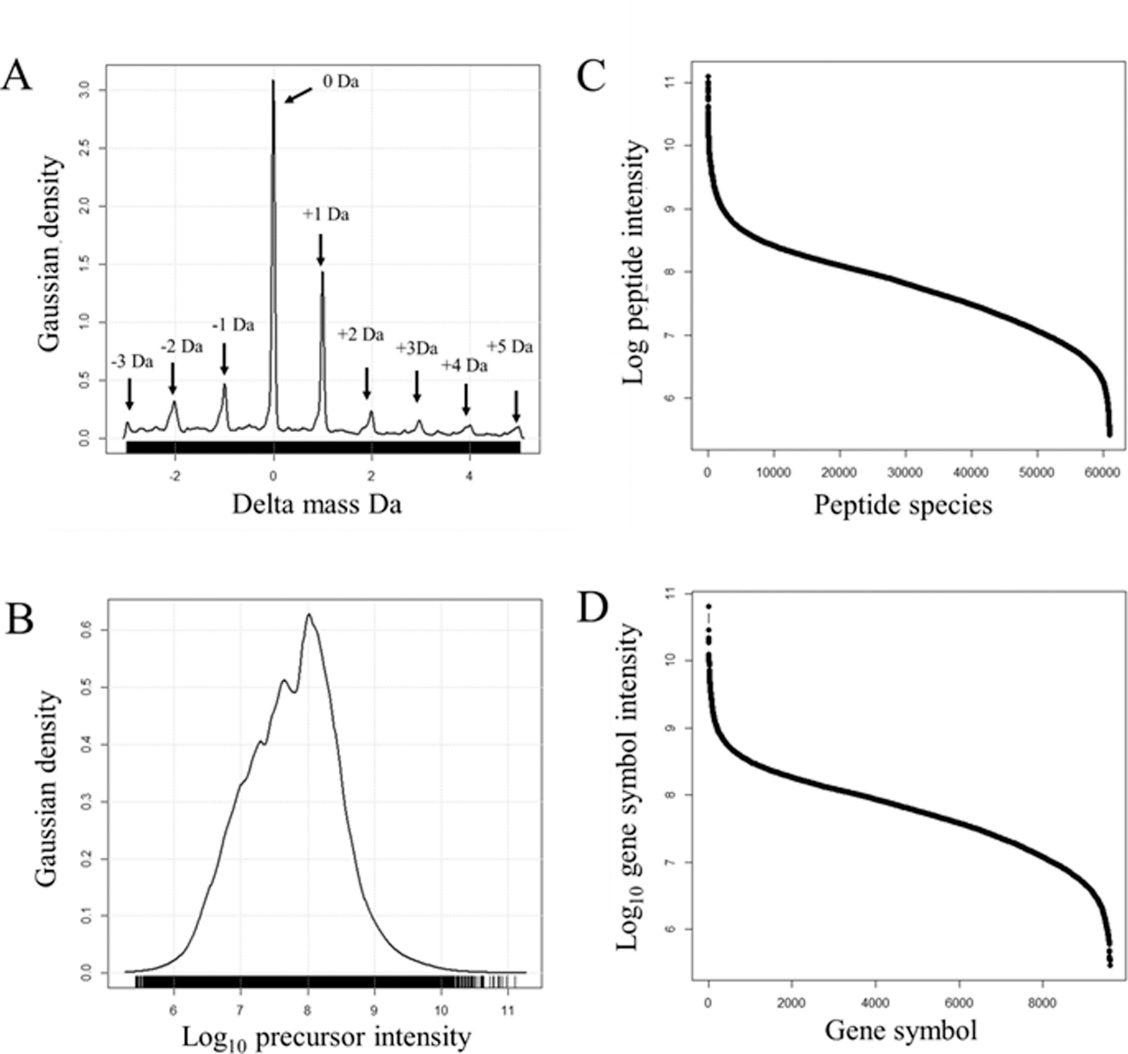
Distributions of the mass accurate results with heavy isotopes and hydrogen rearrangements of MH ± 3 Da showing the delta mass and intensity results of the human plasma protein data from orbital ion trap (OIT). Panels: (A) The delta mass distribution of tryptic peptides from human plasma proteins; (B) The Gaussian log_10_ precursor intensity distribution; (C) The distribution of mean precursor intensity over protein accessions; (D) The distribution of mean precursor intensity over protein gene symbols. The results from 524,867 MS/MS peptide identification events (delta mass −3 to +5 Da) are shown.

The fragmentation spectra (524,867 MS/MS) from the OIT were matched to peptides with a wide mass tolerance and the resulting delta mass distribution was plotted. The OIT data revealed clear peaks in the delta mass distribution at −3, −2, −1, 0, +1, +2, +3, +4, and +5 Da (Figure 2A) which exactly matched the distributions predicted for heavy isotopes and hydrogen rearrangements observed in natural peptides^2,3^ and so is powerful biophysical evidence supporting the veracity of the fit of MS/MS spectra independent of the precursor mass. The precursor intensity distribution was Gaussian from E6 to E10 counts and focused at E8 counts (Figure 2B). The delta mass values were highly concentrated at −3 to +3 Da (Figure 2C). In total, the OIT identified 60,000 peptides from ≥9000 protein gene symbols with wide delta mass distribution (Figure 2D,E) that included peptides with the expected heavy isotope and hydrogen rearrangements. The results indicated that the high energy levels required to resonate in the orbital trap generate hydrogen rearrangements.

### Orbital Ion Trap (OIT) Monoisotopic Mass Distribution

**Figure 3:**
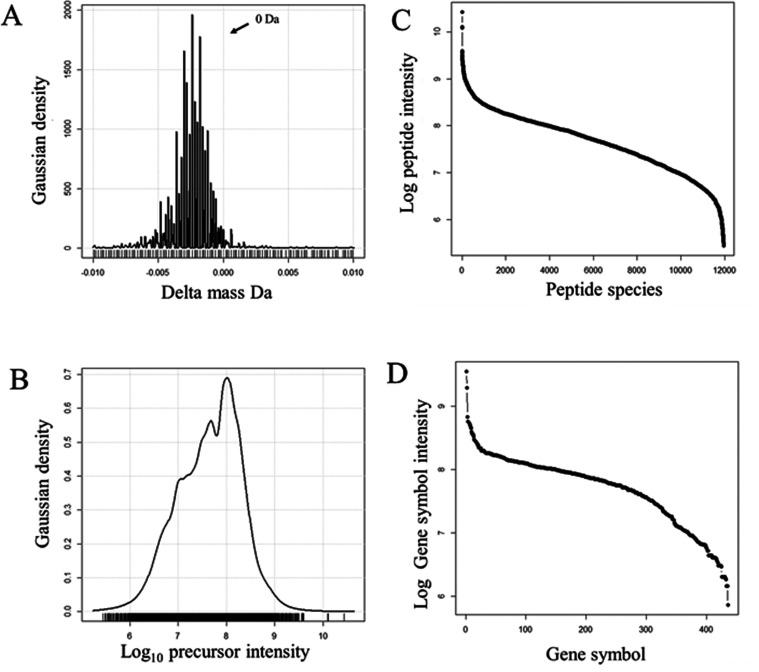
Distributions of the mass accurate data from monoisotopic peptides MH ± 0.1 Da showing the delta mass and intensity results of the human plasma protein data from the OIT. Panels: (A) The distribution of delta mass for tryptic peptides from human plasma proteins; (B) Gaussian log_10_ monoisotopic precursor intensity distribution; (C) The distribution of mean monoisotopic precursor intensities over protein accessions; (D) The distribution of mean monoisotopic precursor intensity over protein gene symbols. Only the monoisotopic results (delta mass −0.1 to +0.1 Da) from a total of 524,867 MS/MS peptide identification events are shown.

The fragmentation spectra (524,867 MS/MS) generated by the OIT instrument displayed distinct peaks within the monoisotopic mass distribution from −0.05 to +0.05 Da (Figure 3A). The precursor intensity distribution was Gaussian from E6 to E10 counts and focused at E8 counts (Figure 3B). Approximately 12,000 peptides (Figure 3C) from 400 human plasma proteins (Figure 3D) were identified at or near the monoisotopic mass. The highly accurate agreement between the predicted precursor mass (MH) and the observations from the OIT clearly demonstrate a low error rate for the analysis of monoisotopic peptides by MS/MS spectra alone.

### Linear Quadrupole Ion Trap (LIT) Delta Mass Distribution

**Figure 4:**
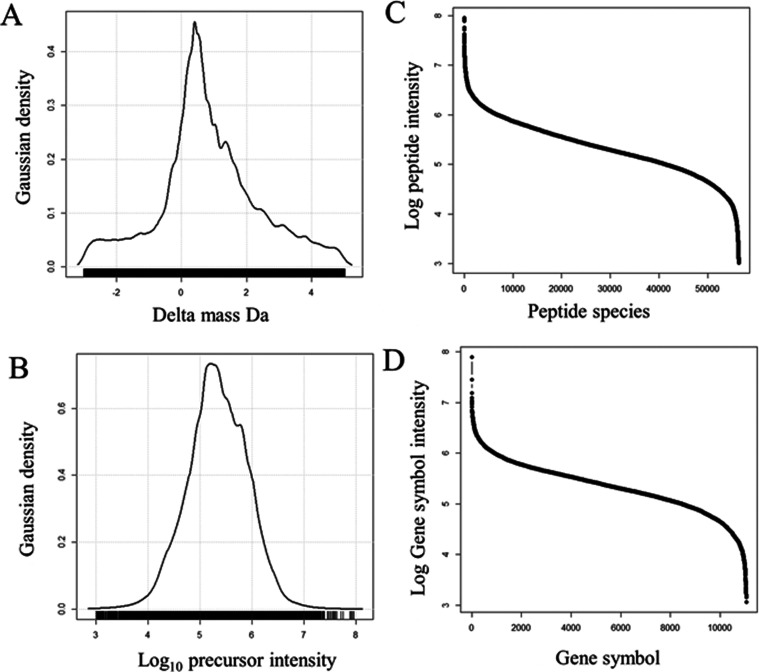
Distributions of the fit of MS/MS spectra in the absence of an accurate mass values (MH ± 3 Da) showing the delta mass and intensity results of the human plasma protein data from the linear quadrupole ion trap (LIT). Panels: (A) The distribution of delta mass for tryptic peptides from human plasma proteins; (B) The Gaussian log_10_ precursor intensity distribution; (C) The distribution of mean precursor intensity over protein accessions; (D) The distribution of mean precursor intensity over protein gene symbols. The results from 1,025,423 MS/MS peptide identification events are shown.

A roughly equal number of identified peptides were obtained using fragmentation spectra (1,025,423 MS/MS) from the LIT that were matched with a wide precursor mass tolerance and the resulting delta mass distributions were plotted. The MS/MS spectra generated by the LIT instrument displayed a delta mass distribution peak from 0 to +2 Da (Figure 4A) that matched the predicted distribution of natural human plasma peptides.^2,3^ The precursor intensity distribution was Gaussian from E4 to E7 counts and focused on E5 counts (Figure 4B). In order to compare the results from MS/MS spectra to those of accurate mass on the basis of equal identified peptides, the LIT was observed to identify a similar number of peptides (∼60,000) (Cf. Figure 2C vs 4C) from 11,000 protein gene symbols from twice as many MS/MS spectra and at ten times the flow rate. The LIT mass analyzer did not seem to generate many peaks from hydrogen rearrangements with the high frequencies observed in spectra generated by the OIT.

### Observation Frequencies from the Fit of Peptide Fragments

**Figure 5:**
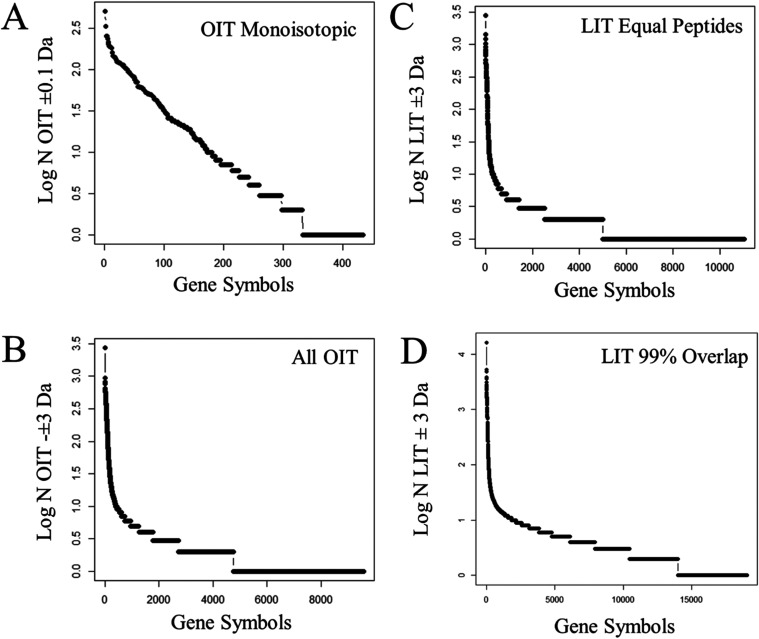
Observation frequencies of protein gene symbols from monoisotopic mass (±0.05 Da) and all delta mass values from the orbital ion trap (OIT) vs the linear quadrupole ion trap (LIT) with different levels of replication. Panels: (A) Peptide observation frequencies of protein gene symbols from the monoisotopic mass (MH ± 0.1 Da) of the OIT (524,867 MS/MS); (B) Peptide observation frequencies of protein gene symbols from all delta mass values (MH ± 3 Da) of the OIT (524,867 MS/MS); (C) Peptide observation frequencies of protein gene symbols from all delta mass values (MH ± 3 Da) of the LIT where the number of peptides identified equaled that of the OIT (1,025,423 MS/MS); (D) Peptide observation frequencies of protein gene symbols from all delta mass values (MH ± 3 Da) of the LIT where there was 99% overlap between the OIT versus LIT (3,786,151 MS/MS).

Observation frequency was computed after selecting the BFPS and correcting for noise with blank analytical control and random MS/MS spectra as statistical control. Peptides identified based on accurate precursor mass (±0.1 Da) from spectra generated by the OIT instrument mapped to 300 protein gene symbols with peptide observations *n* ≥ 3 (Figure 5A). In contrast, accepting the peptides fit from OIT MS/MS spectra with all delta mass values (±3 Da) resulted in the identification of more than 2000 protein gene symbols with *n* ≥ 3 (Figure 5B). Comparing a similar number of peptide identifications from the LIT based on MS/MS fragmentation alone in the absence of an accurate precursor mass also yielded ≥2000 protein gene symbols (Figure 5C). Increasing the sampling of fragment spectra (3,786,151 MS/MS) from the LIT eventually yielded ≥10,000 protein gene symbols (*n* ≥ 3) after correction against analytical and statistical controls (Figure 5D). The maximum accepted protein FDR was ≤1% (*q* ≤ 0.01) false positive rate (i.e. type I error). The OIT monoisotopic mass ±0.01 Da identified 302 proteins gene symbols with *n* ≥ 3 peptides (*q* ≤ 0.01), the OIT ± 3 Da identified 2784 true positive proteins *n* ≥ 3 peptides (*q* ≤ 0.01)^8^ with protein FDR *q*-values ≤0.01 from X!TANDEM. Thus, selecting the strict monoisotopic mass results in about a 90% false negative (type II error) rate compare to accepting all OIT MS/MS from ±3 Da.

### Venn Diagram of Protein Gene Symbols

**Figure 6:**
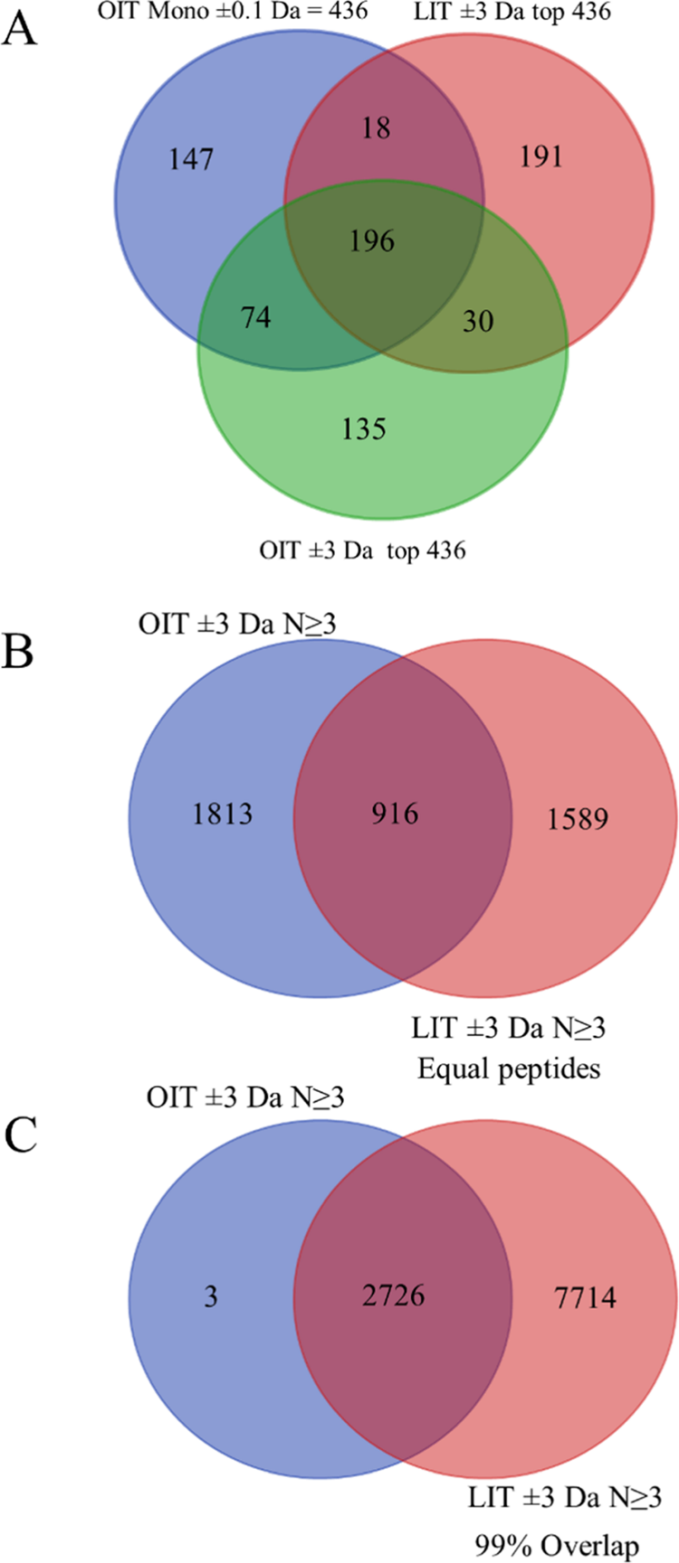
Venn diagrams demonstrate qualitative agreement between the OIT and the LIT at the level of protein gene symbols. Panels: (A) Comparison of an equal number of top-hit proteins. Shown are the top 436 proteins identified from peptides (MH ± 0.01 Da) based on the monoisotopic mass (524,867 MS/MS, in blue), compared to the top 436 proteins of any mass (MH ± 3 Da) from the OIT (in green) and the top 436 proteins from the LIT (in red); (B) Comparison of protein gene symbols from *n* ≥ 3 peptides from an equal number of peptides identified using OIT vs LIT at any delta mass value (MH ± 3 Da); (C) The top 2726 protein gene symbols identified at any mass value (MH ± 3 Da) with *n* ≥ 3 peptides by the OIT vs the sampling required to obtain the same coverage from the LIT (3,786,151 MS/MS).

Analysis of the monoisotopic mass from OIT data yielded at total of 436 proteins; these proteins were then compared to the top 436 proteins identified by the OIT by including all heavy isotopes, hydrogen rearrangements, and other masses and to the top 436 proteins identified from data generated by the LIT analyzer. There was strong agreement on 196 protein gene symbols identified by the OIT (monoisotopic alone and including peptides with other masses) and the LIT (Figure 6A). Also, comparing the fit of fragmentation spectra from the OIT versus the LIT from a similar number of peptides revealed a high level of agreement (Figure 6B). However, the LIT is the more sensitive of the two instruments^24^ and spends most of its cycle time sampling peptides that were below the detection by the OIT. Thus, once the LIT was allowed to sample a sufficient number of peptides, the proteins identified by the instrument were in near-perfect overlap with those identified by the OIT (Figure 6C). The proteins identified by the OIT at the monoisotopic mass showed lower peptide MS/MS observation frequencies (Table 1). The OIT ± 0.1 Da agreed with the OIT ± 3 Da and the LIT ± 3 Da that identified the same peptides from the same proteins in the same proportions consistent with previous observations.^24,25^ However, the MS/MS strategy was more sensitive at the low concentration range and identified some peptides from some proteins that were below detection of the high-resolution instrument and confirmed that plasma peptides and proteins may be identified and quantified from MS/MS spectra alone without the need for an accurate measure of the precursor mass. The maximum accepted protein FDR was ≤1% (*q* ≤ 0.01) false positive rate (type I error). The OIT monoisotopic mass ±0.01 Da identified 302 proteins gene symbols with *n* ≥ 3 peptides (*q* ≤ 0.01), the OIT ± 3 Da identified 2784 true positive proteins *n* ≥ 3 peptides (*q* ≤ 0.01)^8^ with protein FDR *q*-values ≤0.01 from X!TANDEM. Thus, selecting the strict monoisotopic mass results in about a 90% false negative (type II error) rate compared to accepting all OIT MS/MS from ±3 Da.

**Table 1:** Agreement between Results Using the Orbital Ion Trap (OIT) Monoisotopic Mass (OIT mono ±0.1 Da, 524,867 MS/MS) vs OIT and LIT Data That Included Heavy Isotopes, Hydrogen Rearrangements, and Other Masses (OIT ± 3 Da, 524,867 MS/MS; LIT ± 3 Da, 1,025,423 MS/MS)^a^

**gene symbol**	OIT_ ± 0.1 Da (N)	**gene symbol**	OIT ± 3 Da (N)	**gene symbol**	LIT ± 3 Da equal peptide species (N)	**gene symbol**	LIT ± 3 Da 99% gene symbol overlap to OIT (N)
ALB	507	ALB	2743	ALB	2797	ALB	16,405
C3	334	C3	936	APOA1	1434	APOA1	5259
FGA	253	IGH@	824	FGA	1195	TF	4883
CP	252	TF	764	TF	1023	C3	3818
APOA1	236	CP	646	C3	913	IGH@	3665
TF	216	APOA1	641	HP	834	HP	3585
SERPINA1	203	FGA	641	FGB	773	SERPINA1	3098
C4B_2	192	HEL-214	626	CP	730	FGA	2766
C4B	190	SERPINA1	583	IGH@	676	HEL-214	2732
C4A	187	IGHG1	564	FGG	571	IGHG1	2592
IGH@	186	ORM1	538	C4B_2	518	A2M	2361
APOA4	181	HP	515	C4B	512	FGB	2029
FGB	161	IGHG2	487	SERPINA1	508	FLJ00385	1926
ORM1	146	C4B_2	450	C4A	487	IGHG3	1926
ITIH4	143	FGB	447	HEL-214	453	IGHG4	1792
HEL-214	141	C4B	444	HPR	450	IGL@	1783
HP	141	C4A	437	IGHG1	426	HPR	1780
A2M	135	IGL@	432	APOA4	386	CP	1750
FGG	125	IGK@	428	ITIH2	376	C4B_2	1646
AZGP1	122	APOA4	425	A2M	373	C4B	1628
HPX	118	HEL-213	407	IGL@	373	IGHG2	1607
IGHG1	114	ORM2	406	HPX	355	C4A	1587
IGL@	114	AZGP1	395	A1BG	351	APOA4	1549
ORM2	108	FLJ00385	375	SNC73	333	FGG	1542
SERPINC1	100	IGHG3	375	IGHA1	330	HPX	1521
FLJ00385	95	IGHG4	368	IGHG4	316	IGK@	1492
IGHG2	94	A2M	366	FLJ00385	307	HEL-213	1452
IGHG3	94	ITIH4	365	IGHG3	307	APOA2	1397
TTR	93	FGG	337	APOA2	275	SNC73	1300
HPR	90	HPX	323	CFH	264	IGHA1	1279
GC	88	IGKC	295	IgLC-rG	258	IGKC	1265
SERPINA3	86	GC	279	ITIH1	256	IgLC-rG	1254
A1BG	84	A1BG	266	IGHG2	243	IGLC3	1081
HBB	83	SERPINC1	257	IGK@	243	IGLC2	1079
IGK@	82	HBB	252	HEL-213	235	A1BG	1029
HEL-213	81	SNC73	241	IGHA2	227	ITIH2	1021
CLU	76	TTR	232	IGLC2	220	HBB	1004
IGHG4	74	IgLC-rG	230	IGLC3	220	IGHA2	903
CFB	71	IGHA1	224	IGKC	218	AAT	891
ITIH2	71	SERPINA3	220	KNG1	216	IGLL5	814
APCS	70	IGLC3	216	VTN	212	ITIH1	766
ITIH1	62	HPR	214	AHSG	200	ORM1	763
SNC73	62	IGLC2	214	ITIH4	193	ITIH4	755
CFH	61	CFH	204	AAT	164	IGLC6	751
HRG	61	IGLC7	193	IGLL5	162	AHSG	740
IGHM	61	IGHA2	182	GC	159	CFH	732
HBA1	60	ITIH2	181	IGLC7	155	IGLC7	726
HBA2	60	AHSG	176	IGLC6	151	GC	719
KNG1	59	APCS	174	HBB	149	C1	704
AFM	57	ITIH1	172	CLU	135	IGLC1	696

a The LIT ± 3 Da findings show near-perfect agreement with the OIT with respect to protein gene symbols at 3,786,151 MS/MS. The number of peptides identified by the best fit of MS/MS spectra by X!TANDEM after correction against noise and random MS/MS is shown to the right of each protein gene symbol.

### Regression of Peptides to Protein Gene Symbols from Monoisotopic Mass OIT and LIT

**Figure 7:**
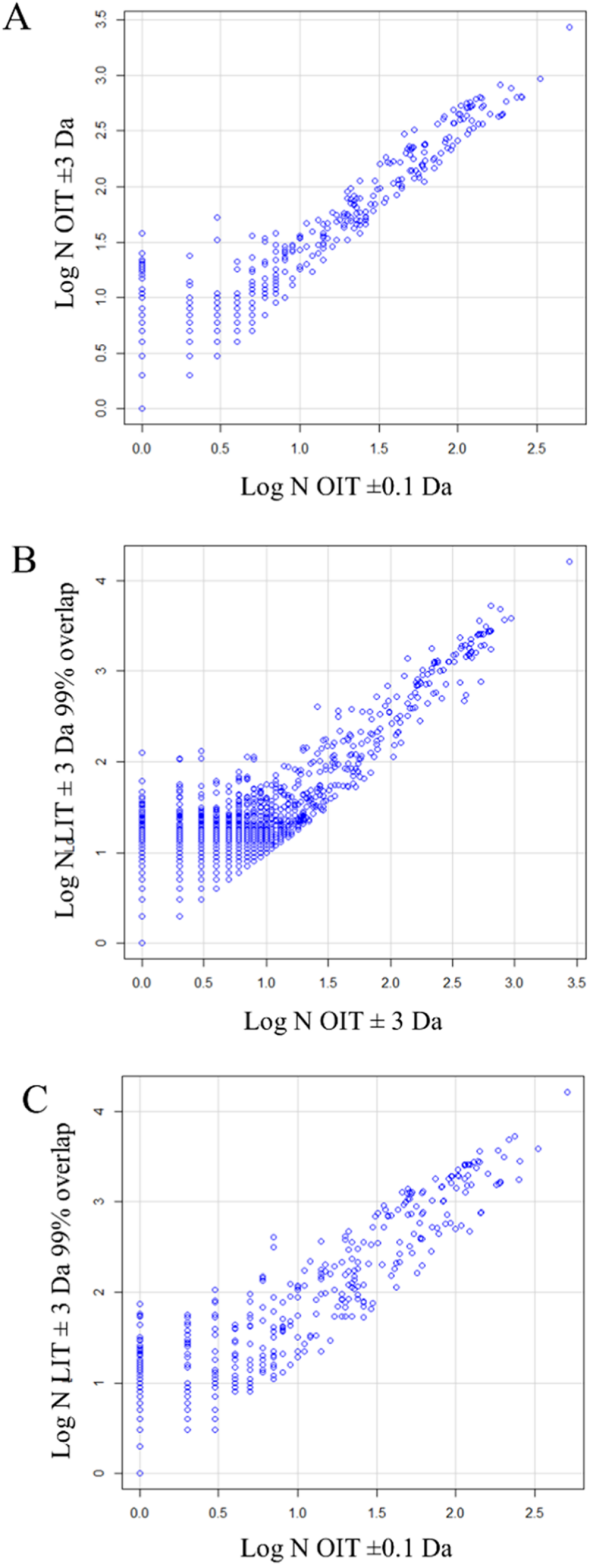
Regression of log-transformed peptide observation frequencies vs protein gene symbols from the LIT onto OIT data. Panels: (A) Regression of all log OIT observations (MH ± 3 Da) onto log of OIT monoisotopic observations (MH ± 0.1 Da) by protein gene symbols (residual standard error: 0.2692 on 433° of freedom (DF), adjusted *R*-squared: 0.8772, F-statistic: 3102 on 1 and 433 DF, *p*-value: < 2.2 × 10^–16^); (B) Regression of all log LIT observations (MH ± 3 Da) onto log OIT observations (MH ± 3 Da) by protein gene symbols (residual standard error: 0.2927 on 9581 DF, adjusted *R*-squared: 0.6003, *F-*statistic: 1.439 × 10^4^ on 1 and 9581 DF, *p*-value: < 2.2 × 10^–16^); (C) Regression of all log LIT observations (MH ± 3 Da) onto log OIT monoisotopic observations (MH ± 0.1 Da) by protein gene symbols (residual standard error: 0.3897 on 433° of freedom, adjusted *R*-squared: 0.8028, *F*-statistic: 1768 on 1 and 433 DF, *p*-value: < 2.2 × 10^–16^).

There was a direct linear relationship between the log of the number of peptides per protein gene symbol identified by each of the two ion trap instruments, demonstrating both qualitative and quantitative agreement between these data sets. The highly significant quantitative relationship between the proteins that were independently identified and counted by the two instruments confirmed the validity of peptide identification based on MS/MS spectra alone within a mass tolerance of ±0.5 Da. Regression of the monoisotopic OIT protein gene symbol observation frequencies onto those determined by including peaks from heavy isotopes and hydrogen rearrangements and all other masses resulted in an adjusted *R-*squared value of 0.8772 with 433 degrees of freedom (DF) that was highly significant (Figure 7A). Regression of all masses from the OIT onto the LIT data resulted in an adjusted *R*-squared of 0.6003 with 9581 DF that was also highly significant (Figure 7B). In addition, regression of the OIT monoisotopic mass data onto all results from the LIT resulted in an *R*-squared value of 0.8028 with 433 DF that was highly significant as well (Figure 7C). The likelihood that all MS/MS spectra generated by each of the two instruments displayed both qualitative and quantitative relationships with the monoisotopic mass data by random chance approached zero. The highly significant qualitative and quantitative agreement between the results of the OIT monoisotopic mass with all other MS/MS spectra also rules out the possibility that analysis of plasma peptides by MS/MS spectra alone had a high error rate. Accepting peptides from the Orbitrap (OIT) at ±3 Da results in more peptides from the same proteins in the same proportion those of the OIT from ±0.1 Da of the monoisotopic that is a powerful biophysical proof of the validity of proteomics [regression *p* < 2.2 × 10^–16^] and shows good agreement with the LIT ± 3 Da. In agreement with previous results true positive peptides of albumin (ALB) from plasma show the same range of peptide *p*-values from false positive peptides of Titin (TTN) from random spectra.^5,6,28,35,36,40,41^ In contrast, the observation frequency of true positive albumin from plasma is high while the observation frequency of false positive ALB from random MS/MS spectra is low and thus the classical Monte Carlo strategy^8,15^ efficiently resolves true positive from false positive results based on observation frequency and subsequent protein *p*-value yet retains excellent sensitivity and so shows great statistical power (Supporting Figures 1–3).

### Agreement at the Level of Peptide Species

**Figure 8:**
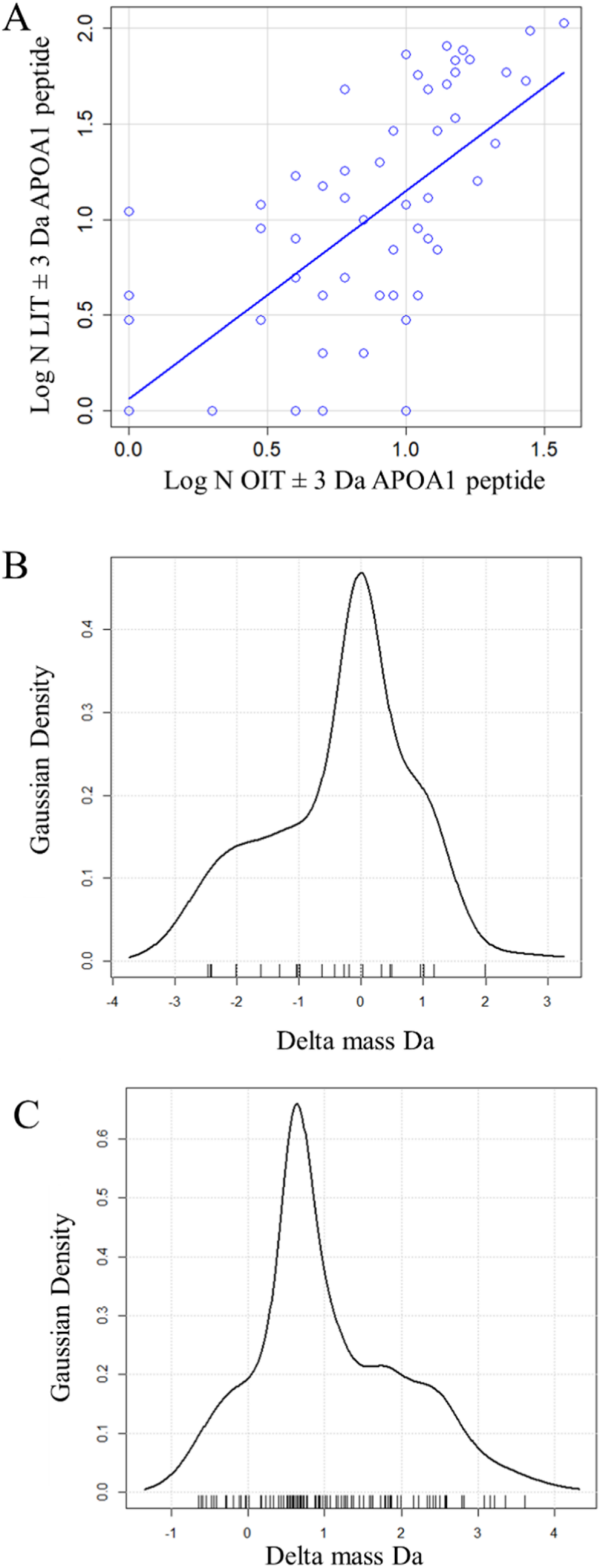
Regression and delta mass density of the common APOA1 peptide, LLDNWDSVTSTFSK, from the OIT vs LIT instrument. (A) Regression of all log LIT peptide observations from APOA1 onto log OIT observations (MH ± 3 Da) over the protein APOA1 (residual standard error: 0.4924 on 54 DF, multiple *R*-squared: 0.3964, F-statistic: 35.46 on 1 and 54 DF, *p*-value = 0.0000002015); (B) Delta mass density from OIT observations of the most common APOA1 peptide; (C) Delta mass density from LIT observations of the most common APOA1 peptide. Regression of the delta mass values from panel C onto those of panel B resulted in a significant *p*-value (2.53 × 10^–14^) and a residual standard error of 0.9096.

The peptides identified by OIT and LIT instruments from the common plasma protein, apolipoprotein (APOA1) were compared across the delta mass distribution. The peptides detected by analysis of the monoisotopic mass peak were also identified at peaks from hydrogen rearrangements, heavy isotopes, and other delta mass values (Supporting Table 1). The same peptides with similar proportions were detected using both the OIT and the LIT instruments (Supporting Table 2). The regression of APOA1 peptide observation frequencies determined by the OIT versus the LIT was highly significant (Figure 8A). The delta mass Gaussian density was centered within 0.5 Da between ± and LIT instruments where the OIT distribution was noticeable skewed toward −3 and −2 Da (Figure 8B,C).

### Plasma Proteins

The accurate monoisotopic peptides compared with the MS/MS spectra from the OIT or LIT without an accurate precursor mass all agreed on the major protein of plasma that should not have occurred if the fit of MS/MS alone showed a large error in peptide identification (Table 1 & Figure 7). The MS/MS from a single quadrupole with the QqQ functions separated in time returned similar identifications and relative observation frequencies of plasma peptides and proteins compared to those obtained using the trihybrid orbital trap from MS/MS or accurate mass. Similarly, the excellent agreement between the MS/MS versus accurate mass with respect to peptide and protein frequencies provided independent evidence that the estimates of peptide identity and observation frequencies made with the robust and sensitive LIT instrument were reliable. The high level of agreement between the lists of plasma proteins identified independently by the OIT and the LIT instrument (after correction with analytical and statistical controls) underscored the low error rate from the fit of fragment spectra alone in the absence of an accurate monoisotopic mass.

## Discussion

The aim of these experiments was to compare the identification of peptides and proteins using accurate precursor mass from the orbitrap (OIT) ± 0.1 Da versus those from the fit of the MS/MS spectra alone from both the OIT ± 3 Da and from the linear quadrupole ion trap (LIT) ± 3 Da. It was critical to first establish that the delta mass distribution of tryptic peptides derived from human plasma proteins using the highly resolving OIT instrument closely agreed with those predicted from heavy isotopes or hydrogen rearrangements. Next it was determined that the set of tryptic peptides that were identified based on monoisotopic mass agreed with those identified across the entire predicted delta mass distribution of the OIT instrument. Finally, it was established that the results of the OIT across the predicted delta mass distribution closely agreed with those from MS/MS spectra alone without an accurate mass estimate from the LIT instrument. The high resolution of the orbital trap was employed to independently confirm that the peptide delta mass distribution matched the predicted heavy isotopes and hydrogen rearrangements within a fraction of a Dalton. Moreover, the peptides and proteins identified from the monoisotopic mass exhibited qualitative and quantitative agreement with those from hydrogen rearrangements, heavy isotopes, and other masses. The human plasma samples recorded by the OIT and the LIT showed excellent agreement with one another at the level of peptides and proteins. The near-perfect agreement (99.9%) of proteins from the OIT versus the LIT unambiguously demonstrated that it was possible to identify peptides from the fit of the MS/MS spectra alone without the need for an accurate precursor mass.^5,6,9^**Recent proteomic analysis of plasma with the orbitrap was only able to detect high abundance molecules like AZGP1, B2M, CRP, HP, HPR, ORM, RBP4, SAA and some others**.^42−52^ In contrast MS/MS spectra in the absence of an accurate precursor mass identified cellular proteins in blood plasma that were undetectable to the orbitrap.^23^

### Delta Mass Distribution of the OIT

The high resolution of the orbital trap revealed that the fit of MS/MS spectra alone correctly identified peptides that matched to the expected delta mass distribution with sharp peaks at the monoisotopic mass as well as at heavy isotopes and hydrogen rearrangements; this information provided powerful biophysical confirmation of the validity of the MS/MS alone for identifying peptides from human plasma proteins. In this study, an analysis of human plasma proteins and their peptides that were identified based on MS/MS fragmentation spectra alone^10^ reliably predicted peptide identity as confirmed by plots of precursor delta mass distribution. Naturally occurring peptides commonly contain heavy isotopes and/or hydrogen rearrangements; the precursor delta mass distribution falls into an integer envelope with expected mass values from −3 to +5 Da.^1−3^ Providing a search tolerance with a wide window (for example, ±3 Da) and then using the high mass resolution of the OIT to determine whether the predicted delta MH values exactly matched the −3, −2, −1, 0, +1, +2, +3, +4, and +5 Da integer distributions expected to result from heavy isotopes and hydrogen rearrangements was a powerful biophysical test of whether the fit of MS/MS spectra alone was sufficient to predict the correct peptide sequence. The OIT provided much greater resolution than the LIT for the measurement of the precursor delta mass values within a small fraction of a Dalton from the monoisotopic or other anticipated integer mass values. Exploiting the high mass resolution of the OIT by searching mass spectrometry data for peptides within ±0.05 Da of the monoisotopic mass leads to a significant loss of sensitivity because most of the data that might be collected from naturally occurring peptides with heavy isotopes, hydrogen rearrangements and other masses will be lost. The hydrogen rearrangement at −1, −2, and −3 as well as the +1 isotope Da has also been apparently observed previously with X!TANDEM and with the Paul trap.^40^

### MS/MS Spectra versus Accurate Precursor Mass

The MS and MS/MS spectra from tryptic peptides of human plasma proteins were recorded using the OIT with the precursor ±0.1 or ±3 Da compared to MS/MS from the LIT without an accurate mass estimate. The OIT is a trihybrid, high-vacuum instrument with mass analyzers separated in space^53^ that delivers high mass resolution albeit with less sensitivity than can be achieved with the linear ion trap.^23,24^ In contrast, the more sensitive LIT^23,24^ performs the precursor scan, trapping, fragmentation, and fragment scan (QqQ) functions using the same low vacuum quadrupole with waveforms separated in time.^54^ Previous comparisons of the LIT to the OIT demonstrated that the two instruments showed near-perfect agreement but that the LIT was more sensitive.^24,25^ The experimentally observed peptide MS/MS spectra from the OIT and LIT may be correlated to theoretical fragmentation spectra of the known human peptides using correlation algorithms such as those found in X!TANDEM.^10^ The MS/MS from the LIT without an accurate precursor mass^19−21^ were sufficient to identify a similar set of peptides, proteins, and gene symbols as the OIT. The predicted delta mass values from the LIT showed the expected distribution of isotopes at 0, +1, and +2 Da as observed. We conclude that the fragment masses ±0.5 Da alone were sufficient to identify many peptides from most plasma proteins using OIT and LIT mass spectrometers. However, there is a temporal cost to running X!TANDEM at a wider mass window where the precursor tolerance of ±0.5 Da required 40 min compute the 9 orbital trap files but required 175 min to compute them with a tolerance of 3 ± Da.

### MS/MS Goodness-of-Fit and Monte Carlo Random MS/MS Corrections

Computing the goodness-of-fit *p*-value with X!TANDEM revealed that most proteins identified with three or more BFPS peptides were reliable^8^ after correcting against the analytical control from blank injections and the random MS/MS spectra statistical control^22,23,55^ before generating the FDR correction using the method of Benjamini and Hochberg.^14^ The classical statistical methods of goodness-of-fit^56^ and Monte Carlo correction^8,15^ against random MS/MS controls^5,6,35,36,41^ confirmed the strong statistical confidence in the peptides identified by MS/MS spectra without a monoisotopic mass from the LIT or OIT instruments.^5,6^ The Monte Carlo correction against noise and random MS/MS spectra followed by calculating the fit of MS/MS spectra to yield *p*-values and FDR *q*-values provided a quantitative assessment of the low error rate from statistical methods. The highly significant regression results revealed a strong linear relation between the peptides identified from the analysis of the monoisotopic mass with those of all the other masses using the OIT and the LIT unambiguously provided highly significant and quantitative evidence of the validity of fitting peptides by the fit of MS/MS spectra alone without the need for an accurate mass estimate. In contrast, the exact match of the delta mass distribution of heavy isotopes and hydrogen rearrangements in the OIT provided direct biophysical evidence for the validity of peptide identification from fragment spectra alone without selecting a monoisotopic mass. The combination of affinity or partition chromatography followed by random and independent sampling^41^ of MS/MS spectra in the absence of an accurate precursor mass for analysis with SQL Server to remove redundant identifications has revealed the ligand receptors from blood cells^57,58^ plasma peptides of disease populations,^59−62^ revealed the growth factors of fetal serum that were confirmed by cellular add-back experiments,^55^ the humoral proteins confirmed by ELISA^22^ and the biomarkers of COVID-19 that were confirmed by enzyme assays.^23^ Biological discoveries using MS/MS spectra from low-resolution ion traps have been confirmed using Western blot,^30^ ELISA^22,33^ enzyme assays^23^ and cellular add-back experiments^55^ as well as specific drugs, genetic mutants, GFP fusions and silencing RNA.^57,58^ We have previously shown that the simple ion trap agrees with the Qq-TOF but was more sensitive.^27−29^

### Agreement at the Level of Peptides and Proteins

Natural peptides show a wide mass distribution;^1−3^ thus, limited sampling to within 0.05 Da of the monoisotopic mass will not provide the highest sensitivity. An analysis across the entire set of peptide delta mass values revealed the peptide species associated with the monoisotopic mass of the OIT were also observed at peaks from heavy isotopes and hydrogen rearrangements and showed qualitative and quantitative agreement with the peptides detected using the LIT instrument. Evidence provided from an analysis of purified peptide or protein standards revealed that many peptides from most proteins can be identified by the MS/MS fragmentation spectra alone^4−7^ and thus that there was no benefit to imposing a narrow precursor mass search window.^9^ Modern 64-bit computers possess more than sufficient power to search the MS/MS spectra over a wider range (±3 Da) so computational efficiency is no longer a major consideration. The validity of fitting peptides by MS/MS alone and the wide natural mass distribution of peptides from −3 to +5 Da provide compelling reasons for the routine adoption of wider search windows which will dramatically increase the analytical power of plasma proteomics by LC-ESI-MS/MS. The strategy of using the best-fit ±3 Da^5,6^ takes into account the existence of naturally occurring heavy isotopes that would present with delta mass values of precisely +1 and +2 Da.^1,2^ Similar changes in delta mass values were observed for potential hydrogen rearrangements of tryptic peptides that may lead to the loss of 1 to 3 Da, as previously reported.^3,40,63^ The most important considerations for rigorous statistical analysis of changes in plasma proteins that may result from different diseases or drug treatments are sensitivity to provide high observation frequencies for confident comparison.^22,23,41,55^ Thus, omitting the strong signals from peptides with heavy isotopes or hydrogen rearrangements represents a large type II error (false negative) that should be avoided to prevent the loss of information of importance to human health. If the fit of MS/MS spectra was false positive then the peptide should have been randomly distributed across the database and the regression should show a flat line with large residuals. Instead, the regression shows the same peptides from the same proteins in the same proportions that could not occur unless and the fit of MS/MS spectra returned the same true positive results as the accurate mass method.

### Human Plasma Proteome

The plasma proteome recorded by the OIT and the LIT instruments showed strong qualitative and quantitative agreement with each other as confirmed by regression analysis.^38,39,64,65^ The data indicated that the MS/MS spectra from human plasma protein digests collected with the LIT or the OIT resulted in at least 2726 protein identifications with nearly perfect qualitative (99.9%) and profound quantitative (*p* ≤ 2 × 10^–16^) agreement. The agreement between the results for known plasma proteins obtained from the independent OIT and LIT instruments also indicated that plasma peptides could be identified and quantified using MS/MS spectra alone. The LIT is a sensitive and robust instrument for the analysis of peptides by LC-ESI-MS/MS where the equations that describe the motion of ions are well understood and their performance can be predicted directly.^54^ The results presented in this study demonstrate that random and independent peptide sampling followed by searching the fragment spectra within ±3 Da for the precursor at a 2+ or 3+ charge state and analyzing fragments from MS/MS spectra within 0.5 Da of the expected theoretical peptide fragmentation pattern are sufficient to identify many peptides from most major plasma proteins with high confidence.^23,41,55^ The results presented here indicate that peptides and proteins in a complex sample like blood plasma can be robustly identified and enumerated using MS/MS spectra in the absence of an accurate precursor ion mass together with a 64-bit computer for classical statistical analysis that delivers a result remarkably similar to those obtained using the accurate precursor mass and has great importance for wide scale application in clinical proteomics.

## Conclusions

The exact match of the isotope distribution and hydrogen rearrangement data identified using the orbital trap provided independent biophysical confirmation of the low error rate of the approach using the best fit of the MS/MS spectra for the identification of human plasma peptides and proteins. The peptides and proteins identified at the monoisotopic mass agreed both qualitatively and quantitatively with the heavy isotope and hydrogen rearrangement data. The best fit of the MS/MS spectra from the OIT included the same peptides and proteins as identified using the LIT. In this study, independent lines of evidence from the biophysical agreement of predicted and measured monoisotopic mass, the exact delta mass distribution of hydrogen rearrangements and heavy isotopes, the fit of MS/MS to peptide *p*-values and FDR corrected *q*-values, the Monte Carlo correction against noise and 30,000,000 random MS/MS spectra, the qualitative agreement shown by Venn diagram, and the quantitative agreement determined by regression all demonstrated the low error rate of the OIT and excellent agreement with the LIT without specifying an accurate precursor mass. It is important to note that, after calculating the predicted precursor monoisotopic mass from the best-fit of the MS/MS spectra, the delta mass values independently exhibited the predicted peaks at exactly −3, −2, −1, 0, +1, +2, +3, +4, and +5 Da.^3^ The excellent fit to the expected delta mass distribution provides powerful biophysical evidence for the validity of peptide identification from goodness of fit using the MS/MS spectra alone. The delta mass and statistical results demonstrated that many peptides from most plasma proteins can be identified and enumerated using the sensitive fit of MS/MS spectra without an accurate monoisotopic peptide mass.^22,23,55^ In agreement with previous studies,^5,6^ the largest sources of error in proteomics were not from the mass estimates or the instruments employed but were from computational sources that could be corrected by removing redundancy with an SQL Server and correcting with noise analytical and random MS/MS statistical controls in the R statistical system.^5,6^ Here the comparison of the human plasma peptides from the fit of fragmentation spectra provided nearly identical results to the accurate monoisotopic precursor mass but the MS/MS approach was much more sensitive. Taken together this paper demonstrates that combining standard precipitation with convention protein chromatography followed by simple trypsin digestion and MS/MS analysis with robust and sensitive linear ion traps may provide a large plasma proteome with low Type I and Type II error using only classical statistical techniques with the widely available SQL Server and R statistical system that has profound implications for the clinical analysis of blood samples.

## Materials and Methods

### Materials

The Dionex UltiMate 3000 series ultraperformance liquid chromatography (UPLC), C18 Acclaim PepMap NanoLC column (75 μm ID, 25 cm length C18), and Fusion Lumos (Q-Orbital ion trap-LTQ Tribrid MS) were obtained from Thermo Fisher Scientific (Waltham, MA). The Hewlett-Packard 1100 high-performance liquid chromatography (HPLC) (Santa Clara, CA) was coupled to an LTQ XL linear ion trap mass spectrometer (Thermo Electron Corporation, Madison, WI). The salts, buffers, and Coomassie Blue were obtained from Sigma-Aldrich (St. Louis, MO). The QA resin on a ceramic support was from Bio-Rad (Hercules, CA). HPLC-grade water, ethanol, acetone, and acetonitrile (ACN) were obtained from Caledon Laboratories (Georgetown, Ontario, Canada). The analytical C18 resin was Princeton Sphere 5 μm 300 Å (Princeton Chromatography, Cranbury, NJ). Sequencing grade trypsin was from Promega (Madison, Wisconsin).

### Plasma Separation and Analysis

Plasma samples (25 μL) were precipitated with 10 volumes of ACN,^31^ and centrifuged at 12,000 relative centrifugal force (RCF) for 15 min at room temperature and the pellet dried under vacuum. The proteins were resuspended in 250 μL of 20 mM pH 8.85 tris buffer on ice after vortexing and then centrifuged at 12,000 RCF for 5 min; the supernatant was then collected. The intact plasma proteins were collected over 100 μL of **Quaternary amine (QA) also called Strong Anion Exchange (SAX) resin or simply Q resin**, washed with three column volumes, and eluted with two column volumes (200 μL) of ≤1 M NaCl.^34^ The resulting protein samples (200 μL) were digested in 600 mM urea and 5% ACN in 20 mM tris pH 8.85 with trypsin at 1/100 at 37 °C for ≤16 h, reduced with 2 mM dithiothreitol at 50 °C for 20 min, and digested again with trypsin for 2h.^34^

### C18 ZipTips

The plasma peptides were collected over a C18 ZipTip column in 5% acetic acid, washed, and eluted in 2 μL 5% formic acid, and 65% ACN. The eluate was diluted immediately with 18 μL of 5% formic acid for injection via a 20 μL loop.

### Linear Quadrupole Ion Trap (LIT)

Analytical HPLC was performed over 90 min with a 5 to 65% ACN gradient on a 150-μm ID column (15 cm) with in-line filter frits as previously described.^22^ The peptides were ionized by nano spray of the solvent gradient at 2 μL/min by a 1:10 split to a flow of ∼200 nL/min with a transfer capillary temperature of 250 °C in a Thermo Electron Corporation LTQ XL ion trap mass spectrometer.^54^ Approximately 5 μg of extracted and purified peptides were injected via a 20 μL sample loop. The electrospray voltage was 1500 v and the instrument was set to sample the 5 most intense ions randomly with respect to time^41^ as they eluted with up to 200 ms to fill the trap to a target 250,000 ions.

### Orbital Ion Trap (OIT)

The plasma peptides collected over a C18 ZipTip were eluted in 2 μL of 5% formic acid and 65% ACN and immediately diluted with 18 μL of 5% formic acid for injections via a 20 μL loop. The resulting peptides were analyzed over an Acclaim PepMap Nano LC column (C18, 2 μm particles with a pore size of 100 Å, ID: 0.075 mm × 250 mm) at 30 nL per minute with a 50 min gradient from 5 to 70% acetonitrile for the Thermo orbital ion trap, Fusion Lumos (Q-Orbital ion trap-LTQ Tribrid MS) at the Department of Chemistry, National Taiwan University. The instrument was tested with the manufacturer’s calibration mixture. The electrospray was formed at 2000 V and the ion transfer tube was maintained at 275 °C. The tryptic peptides were identified by MS scans from *m*/*z* 350 to *m*/*z* 1700 and followed by higher-energy collisional dissociation-MS/MS spectra of the most intense ions at a normalized collision energy of 32%. The MS survey scan was performed every 3 s (AGC target 5e5, maximum injection time of 50 ms) and all ions above the signal threshold were submitted for MS/MS (AGC target 5e4, maximum injection time of 50 ms) in order of decreasing intensity with previously selected ions dynamically excluded for 60s and the isolation width was 1.4 Th

### Peptide MS/MS Spectra Correlation Analysis

A physical filter of at least 1000 (E3) intensity counts for peptide parent ions was used to limit type I error.^22^ The MS/MS spectra were fit to human peptides cataloged in the UniParc protein library^66^ as of 2023. The MS/MS spectra were fit to peptides in the absence of an accurate precursor mass by the X!TANDEM algorithm which generates *p*-values directly from the fit of predicted versus observed MS/MS spectra by the standard statistical method of goodness of fit.^10,12,67−69^ Fully tryptic enzyme specifications that included a charge state of 2+ or 3+, as many as three missed cleavages, and a wide precursor mass tolerance (±3 Da) were used to calculate the peptide [M + H]^+^ and fragments within 0.5 Da.^5,6,40^ Protein counts from the best-fit per MS/MS spectra (BFPS) were computed in the SQL Server with each MS/MS spectra used only once and assigned to the best-fit peptide for the computation of protein (gene symbol) *p*-values and FDR corrected *q*-values.^14^ The MS/MS spectra were fit to peptides with fully tryptic X!TANDEM settings.^10^ The X!TANDEM algorithm was set to search parameters of 2+ or 3+ precursor ions from 500 to 2000 *m*/*z* that correlated from −3 to +5 Da with fragments ±0.5 Da and a maximal accepted value of *p* ≤ 0.01.^5,6^ TRYP [RK]|[X] conditions included peptides with modifications such as the oxidation of methionine (M) or tryptophan (W) (15.994915), the addition of +1 at asparagine (N) or glutamine (Q) (0.984016), and hydroxylation or sulfonation of M or W (31.98983) and glycine (G) substitution at Cysteine (C) (57.021464). The addition of a carboxy-terminal hydroxyl (17.002735) or an amino-terminal proton (1.007825) was also included with a maximum protein *p*-value of *p* ≤ 0.01. The X!TANDEM algorithm^10^ provided a computed *p*-value for each peptide match together with an estimate of type I error rate.^5,6,41^

### Random MS/MS Control

Random MS/MS spectra were created using a modification of the random MS/MS spectra generator as previously described.^5,6^ The range of precursor masses, the range and number of fragments and their mass distribution were matched to those of the experimental observations. The precursors were *z* = +2 to z = +3 and ranged from 350 to 2000 *m*/*z* (i.e., 700 to 6000 Da). Fragments were restricted to z = +1 with 150 m/*z* ≤ 2000 Da.

### Computational Analysis in SQL and Statistical Analysis with R

The LC-ESI-MS/MS spectra, including the parent and fragment *m*/*z* and intensity values, together with the resulting peptide and protein identifications from the X!TANDEM,^10^ were introduced into an SQL Server database.^39^ The inherent features of the SQL Server system permitted the MS/MS spectra to be assigned to a nonredundant set of BFPSs. The experimental results were collected and corrected with analytical controls that included blank injection runs with HPLC-grade solvents over untreated columns.^5,6,40^ The observation frequencies of peptide species from the experimental recordings were resolved from those of blank injection LC-ESI-MS/MS recordings and random MS/MS spectra by the chi Square test (χ^2^ ≥ 9, *p* ≤ p0.01). The LC-ESI-MS/MS data were graphically and statistically analyzed by regression with the R statistical system.^41^ The R statistical system was used to plot data density and distribution and to compute the cumulative *p*-value for protein gene symbols with FDR correction to *q*-values by the method of Benjamini and Hochberg.^14^ The plasma samples resolved by the LIT were compared to those of the OIT under three conditions, specifically, (1) an equal number of proteins identified by each instrument; (2) a roughly equal number of peptides identified by each instrument, and (3) sufficient sampling with the LIT to achieve nearly complete overlap with the OIT instrument.

## Data Availability

The computed results are provided in the Supporting data. The data from the SQL Server database is available upon request. Raw data files are available via ProteomeXchange with identifier PXD055594 and PXD055704.

## Supporting Information Available

The characteristics of the common APOA1 peptide, LLDNWDSVTSTFSK from the orbital ion trap; The characteristics of the common APOA1 peptides from the LIT instrument versus the OIT instrument from the fit of MS/MS spectra; The false positive peptide *p*-value and peptide log *p*-value distribution of all random MS/MS spectra and those of Albumin (ALB) and Titan (TTN);The peptide *p*-value and peptide log *p*-value distribution from uncorrected OIT MS/MS spectra ±3 Da versus those of Albumin (ALB) and Titan (TTN); The peptide *p*-value and gene symbol *p*-value distribution and log(*n*) distribution from all OIT MS/MS spectra ±3 Da after noise and random MS/MS correction versus those of the accurate monoisotopic mass only and the true positive plasma proteins (PDF)

## Notes

Normal plasma samples for LIT analysis were obtained from the International Biobank of Luxembourg of the Luxembourg Institute of Health (LIH) collected under a Comité National d’Ethique de Recherche (CNER) Protocol 201107/02 “Biospecimen Research” at the Centre Hospitalier de Luxembourg. There was no ethical approval required for the commercial plasma used for the OIT experiments.

## Abbreviations

ACN: acetonitrile
APOA1: apolipoprotein A1
BFPS: best-fit per MS/MS spectra to tryptic peptides
DF: degrees of freedom
FTICR: Fourier-transform ion cyclotron resonance
HPLC: high-performance liquid chromatography
LC-ESI-MS/MS: liquid chromatography nano electrospray tandem mass spectrometry
LIT: linear quadrupole ion trap
MH: predicted precursor mass [M + H]^+^
OIT: orbital ion trap
PPI: protein–protein interactions
QA: quaternary amine
QqQ-TOF: triple quadrupole-time-of-flight
RCF: relative centrifugal force
UPLC: ultraperformance liquid chromatography

## References

[ref1] Kantnerová K.; Kuhlbusch N.; Juchelka D.; et al. A guide to precise measurements of isotope abundance by ESI-Orbitrap MS. Nat. Protoc. 2024, 19, 2435–2466. 10.1038/s41596-024-00981-5. 38654136

[ref2] Popovic Z.; Anderson L. C.; Zhang X.; et al. Analysis of Isotopically Depleted Proteins Derived from *Escherichia coli* and Caenorhabditis elegans Cell Lines by Liquid Chromatography 21 T Fourier Transform-Ion Cyclotron Resonance Mass Spectrometry. J. Am. Soc. Mass Spectrom. 2023, 34 (2), 137–144. 10.1021/jasms.2c00242. 36656140

[ref3] Savitski M. M.; Kjeldsen F.; Nielsen M. L.; et al. Hydrogen rearrangement to and from radical z fragments in electron capture dissociation of peptides. J. Am. Soc. Mass Spectrom. 2007, 18 (1), 113–120. 10.1016/j.jasms.2006.09.008. 17059886

[ref4] Guzzetta A. W.; Thakur R. A.; Mylchreest I. C. A robust micro-electrospray ionization technique for high-throughput liquid chromatography/mass spectrometry proteomics using a sanded metal needle as an emitter. Rapid Commun. Mass Spectrom. 2002, 16 (21), 2067–2072. 10.1002/rcm.829. 12391582

[ref5] Dufresne J.; Florentinus-Mefailoski A.; Zhu P. H.; et al. Re-evaluation of the rabbit myosin protein standard used to create the empirical statistical model for decoy library searching. Anal. Biochem. 2018, 560, 39–49. 10.1016/j.ab.2018.08.025. 30171831

[ref6] Thavarajah T.; Tucholska M.; Zhu P. H.; et al. Re-evaluation of the 18 non-human protein standards used to create the Empirical Statistical Model for Decoy Library Searching. Anal. Biochem. 2020, 599, 113680 10.1016/j.ab.2020.113680. 32194076

[ref7] Zolg D. P.; Wilhelm M.; Schnatbaum K.; et al. Building ProteomeTools based on a complete synthetic human proteome. Nat. Methods 2017, 14 (3), 259–262. 10.1038/nmeth.4153. 28135259 PMC5868332

[ref8] Cargile B. J.; Bundy J. L.; Stephenson J. L. Jr. Potential for false positive identifications from large databases through tandem mass spectrometry. J. Proteome Res. 2004, 3 (5), 1082–1085. 10.1021/pr049946o. 15473699

[ref9] Chick J. M.; Kolippakkam D.; Nusinow D. P.; et al. A mass-tolerant database search identifies a large proportion of unassigned spectra in shotgun proteomics as modified peptides. Nat. Biotechnol. 2015, 33 (7), 743–749. 10.1038/nbt.3267. 26076430 PMC4515955

[ref10] Craig R.; Beavis R. C. TANDEM: matching proteins with tandem mass spectra. Bioinformatics 2004, 20 (9), 1466–1467. 10.1093/bioinformatics/bth092. 14976030

[ref11] Eng J. K.; McCormack A. L.; Yates J. R. An approach to correlate tandem mass spectral data of peptides with amino acid sequences in a protein database. J. Am. Soc. Mass Spectrom. 1994, 5 (11), 976–989. 10.1016/1044-0305(94)80016-2. 24226387

[ref12] Field H. I.; Fenyo D.; Beavis R. C. RADARS, a bioinformatics solution that automates proteome mass spectral analysis, optimizes protein identification, and archives data in a relational database. Proteomics 2002, 2 (1), 36–47. 10.1002/1615-9861(200201)2:1<36::AID-PROT36>3.0.CO;2-W. 11788990

[ref13] Moore R. E.; Young M. K.; Lee T. D. Qscore: an algorithm for evaluating SEQUEST database search results. J. Am. Soc. Mass Spectrom. 2002, 13 (4), 378–386. 10.1016/S1044-0305(02)00352-5. 11951976

[ref14] Benjamini Y.; Hochberg Y. Controlling false discovery rate: A practical approach to multiple testing. J. R. Stat. Soc., Ser. B 1995, 57 (1), 289–300. 10.1111/j.2517-6161.1995.tb02031.x.

[ref15] Hope A. C. A. A Simplified Monte Carlo Significance Test Procedure. J. R. Stat. Soc., Ser. B 1968, 30 (3), 582–598. 10.1111/j.2517-6161.1968.tb00759.x.

[ref16] Martínez A. C.; Solis A.; Díaz Hernández Rojas R.; et al. Advanced Statistical Testing of Quantum Random Number Generators. Entropy 2018, 20 (11), 886 10.3390/e20110886. 33266609 PMC7512468

[ref17] Park S. K.; M K. Random Number Generators: Good Ones Are Hard To Find. Commun. ACM 1988, 31 (10), 1192–1201. 10.1145/63039.63042.

[ref18] Rice S. O. Mathematical Analysis of Random Noise. Bell Syst. Tech. J. 1945, 24, 46–156. 10.1002/j.1538-7305.1945.tb00453.x.

[ref19] Washburn M. P.; Wolters D.; Yates J. R. 3rd Large-scale analysis of the yeast proteome by multidimensional protein identification technology. Nat. Biotechnol. 2001, 19 (3), 242–247. 10.1038/85686. 11231557

[ref20] Link A. J.; Eng J.; Schieltz D. M.; et al. Direct analysis of protein complexes using mass spectrometry. Nat. Biotechnol. 1999, 17 (7), 676–682. 10.1038/10890. 10404161

[ref21] McCormack A. L.; Schieltz D. M.; Goode B.; et al. Direct analysis and identification of proteins in mixtures by LC/MS/MS and database searching at the low-femtomole level. Anal. Chem. 1997, 69 (4), 767–776. 10.1021/ac960799q. 9043199

[ref22] Chen Z. Z.; Dufresne J.; Bowden P.; et al. Extraction of naturally occurring peptides versus the tryptic digestion of proteins from fetal versus adult bovine serum for LC-ESI-MS/MS. Anal. Biochem. 2024, 689, 115497 10.1016/j.ab.2024.115497. 38461948

[ref23] Chen Z. Z.; Johnson L.; Trahtemberg U.; et al. Mitochondria and cytochrome components released into the plasma of severe COVID-19 and ICU acute respiratory distress syndrome patients. Clin. Proteomics 2023, 20 (1), 17 10.1186/s12014-023-09394-0. 37031181 PMC10082440

[ref24] Wong C. C. L.; Cociorva D.; Venable J. D.; et al. Comparison of different signal thresholds on data dependent sampling in Orbitrap and LTQ mass spectrometry for the identification of peptides and proteins in complex mixtures. J. Am. Soc. Mass Spectrom. 2009, 20 (8), 1405–1414. 10.1016/j.jasms.2009.04.007. 19467883 PMC2946190

[ref25] Heil L. R.; Remes P. M.; MacCoss M. J. Comparison of Unit Resolution Versus High-Resolution Accurate Mass for Parallel Reaction Monitoring. J. Proteome Res. 2021, 20 (9), 4435–4442. 10.1021/acs.jproteome.1c00377. 34319745

[ref26] Omenn G. S.; States D.; Adamski M.; et al. Overview of the HUPO Plasma Proteome Project: results from the pilot phase with 35 collaborating laboratories and multiple analytical groups, generating a core dataset of 3020 proteins and a publicly-available database. Proteomics 2005, 5 (13), 3226–3245. 10.1002/pmic.200500358. 16104056

[ref27] Marshall J.; Jankowski A.; Furesz S.; et al. Human serum proteins preseparated by electrophoresis or chromatography followed by tandem mass spectrometry. J. Proteome Res. 2004, 3 (3), 364–382. 10.1021/pr034039p. 15253417

[ref28] Tucholska M.; Florentinus A.; Williams D.; et al. The endogenous peptides of normal human serum extracted from the acetonitrile-insoluble precipitate using modified aqueous buffer with analysis by LC-ESI-Paul ion trap and Qq-TOF. J. Proteomics 2010, 73 (6), 1254–1269. 10.1016/j.jprot.2010.02.022. 20211283

[ref29] Williams D.; Ackloo S.; Zhu P.; et al. Precipitation and selective extraction of human serum endogenous peptides with analysis by quadrupole time-of-flight mass spectrometry reveals posttranslational modifications and low-abundance peptides. Anal. Bioanal. Chem. 2010, 396, 1223–1247. 10.1007/s00216-009-3345-0. 20033139

[ref30] Marshall J.; Kupchak P.; Zhu W.; et al. Processing of serum proteins underlies the mass spectral fingerprinting of myocardial infarction. J. Proteome Res. 2003, 2, 361–372. 10.1021/pr030003l. 12938926

[ref31] Tucholska M.; Scozzaro S.; Williams D.; et al. Endogenous peptides from biophysical and biochemical fractionation of serum analyzed by matrix-assisted laser desorption/ionization and electrospray ionization hybrid quadrupole time-of-flight. Anal. Biochem. 2007, 370, 228–245. 10.1016/j.ab.2007.07.029. 17884004

[ref32] Dufresne J.; Florentinus-Mefailoski A.; Bowden P.; et al. A method for the extraction of the endogenous tryptic peptides (peptidome) from human EDTA plasma. Anal. Biochem. 2018, 549, 188–196. 10.1016/j.ab.2018.02.025. 29486203

[ref33] Zhang R.; Barker L.; Pinchev D.; et al. Mining biomarkers in human sera using proteomic tools. Proteomics 2004, 4 (1), 244–256. 10.1002/pmic.200300495. 14730686

[ref34] Tucholska M.; Bowden P.; Jacks K.; et al. Human serum proteins fractionated by preparative partition chromatography prior to LC-ESI-MS/MS. J. Proteome Res. 2009, 8, 1143–1155. 10.1021/pr8005217. 19265436

[ref35] Zhu P.; Bowden P.; Tucholska M.; et al. Chi-square comparison of tryptic peptide-to-protein distributions of tandem mass spectrometry from blood with those of random expectation. Anal. Biochem. 2011, 409 (2), 189–194. 10.1016/j.ab.2010.10.027. 20977879

[ref36] Zhu P.; Bowden P.; Tucholska M.; et al. Peptide-to-protein distribution versus a competition for significance to estimate error rate in blood protein identification. Anal. Biochem. 2011, 411, 241–253. 10.1016/j.ab.2010.12.003. 21138726

[ref37] Peihong Zhu P. B.; Pendrak V.; Thiele H.; Zhang D.; Siu M.; Diamandis E. P.; Marshall J. Comparison of Protein Expression Lists From Mass Spectrometry of Human Blood Fluids Using Exact Peptide Sequences Versus BLAST. Clin. Proteomics 2007, 2 (1), 185–203.

[ref38] Bowden P.; Pendrak V.; Zhu P.; et al. Meta sequence analysis of human blood peptides and their parent proteins. J. Proteomics 2010, 73, 1163–1175. 10.1016/j.jprot.2010.02.007. 20170764

[ref39] Bowden P.; Beavis R.; Marshall J. Tandem mass spectrometry of human tryptic blood peptides calculated by a statistical algorithm and captured by a relational database with exploration by a general statistical analysis system. J. Proteomics 2009, 73, 103–111. 10.1016/j.jprot.2009.08.004. 19703602

[ref40] Florentinus A. K.; Bowden P.; Sardana G.; et al. Identification and quantification of peptides and proteins secreted from prostate epithelial cells by unbiased liquid chromatography tandem mass spectrometry using goodness of fit and analysis of variance. J. Proteomics 2012, 75, 1303–1317. 10.1016/j.jprot.2011.11.002. 22120120

[ref41] Dufresne J.; Florentinus-Mefailoski A.; Ajambo J.; et al. Random and independent sampling of endogenous tryptic peptides from normal human EDTA plasma by liquid chromatography micro electrospray ionization and tandem mass spectrometry. Clin. Proteomics 2017, 14, 41 10.1186/s12014-017-9176-7. 29234243 PMC5721679

[ref42] Kimhofer T.; Lodge S.; Whiley L.; et al. Integrative Modeling of Quantitative Plasma Lipoprotein, Metabolic, and Amino Acid Data Reveals a Multiorgan Pathological Signature of SARS-CoV-2 Infection. J. Proteome Res. 2020, 19 (11), 4442–4454. 10.1021/acs.jproteome.0c00519. 32806897

[ref43] Wan J.; Sun W.; Li X.; et al. Inflammation inhibitors were remarkably up-regulated in plasma of severe acute respiratory syndrome patients at progressive phase. Proteomics 2006, 6 (9), 2886–2894. 10.1002/pmic.200500638. 16649161 PMC7168070

[ref44] Giordano A.; Spagnolo V.; Colombo A.; et al. Changes in some acute phase protein and immunoglobulin concentrations in cats affected by feline infectious peritonitis or exposed to feline coronavirus infection. Vet J. 2004, 167 (1), 38–44. 10.1016/S1090-0233(03)00055-8. 14623149 PMC7128346

[ref45] Ponti G.; Maccaferri M.; Ruini C.; et al. Biomarkers associated with COVID-19 disease progression. Crit. Rev. Clin. Lab. Sci. 2020, 57 (6), 389–399. 10.1080/10408363.2020.1770685. 32503382 PMC7284147

[ref46] Elshazli R. M.; Toraih E. A.; Elgaml A.; et al. Diagnostic and prognostic value of hematological and immunological markers in COVID-19 infection: A meta-analysis of 6320 patients. PLoS One 2020, 15 (8), e0238160 10.1371/journal.pone.0238160. 32822430 PMC7446892

[ref47] Schultze J. L.; Aschenbrenner A. C. COVID-19 and the human innate immune system. Cell 2021, 184 (7), 1671–1692. 10.1016/j.cell.2021.02.029. 33743212 PMC7885626

[ref48] Overmyer K. A.; Shishkova E.; Miller I. J.; et al. Large-Scale Multi-omic Analysis of COVID-19 Severity. Cells Syst. 2021, 12 (1), 23–40 e7. 10.1016/j.cels.2020.10.003. PMC7543711 33096026

[ref49] Thompson E. A.; Cascino K.; Ordonez A. A.; et al. Metabolic programs define dysfunctional immune responses in severe COVID-19 patients. Cell Rep. 2021, 34 (11), 108863 10.1016/j.celrep.2021.108863. 33691089 PMC7908880

[ref50] Lee J. S.; Han D.; Kim S. Y.; et al. Longitudinal proteomic profiling provides insights into host response and proteome dynamics in COVID-19 progression. Proteomics 2021, 21 (11–12), e2000278 10.1002/pmic.202000278. 33945677 PMC8206655

[ref51] Park J.; Kim H.; Kim S. Y.; et al. In-depth blood proteome profiling analysis revealed distinct functional characteristics of plasma proteins between severe and non-severe COVID-19 patients. Sci. Rep. 2020, 10 (1), 22418 10.1038/s41598-020-80120-8. 33376242 PMC7772338

[ref52] Shu T.; Ning W.; Wu D.; et al. Plasma Proteomics Identify Biomarkers and Pathogenesis of COVID-19. Immunity 2020, 53 (5), 1108–1122 e5. 10.1016/j.immuni.2020.10.008. 33128875 PMC7574896

[ref53] Makarov A. Electrostatic axially harmonic orbital trapping: a high-performance technique of mass analysis. Anal. Chem. 2000, 72 (6), 1156–1162. 10.1021/ac991131p. 10740853

[ref54] Schwartz J. C.; Senko M. W.; Syka J. E. A two-dimensional quadrupole ion trap mass spectrometer. J. Am. Soc. Mass Spectrom. 2002, 13 (6), 659–669. 10.1016/S1044-0305(02)00384-7. 12056566

[ref55] Chen Z. Z.; Bowden P.; Dufresne J.; et al. LEDGF is a new growth factor in fetal serum. Anal. Biochem. 2022, 655, 114845 10.1016/j.ab.2022.114845. 35970411

[ref56] Bowden P.; Thavarajah T.; Zhu P.; et al. Quantitative statistical analysis of standard and human blood proteins from liquid chromatography, electrospray ionization, and tandem mass spectrometry. J. Proteome Res. 2012, 11, 2032–2047. 10.1021/pr2000013. 22316523

[ref57] Jankowski A.; Zhu P.; Marshall J. G. Capture of an activated receptor complex from the surface of live cells by affinity receptor chromatography. Anal. Biochem. 2008, 380, 235–248. 10.1016/j.ab.2008.05.047. 18601892

[ref58] Howard J. C.; Florentinus-Mefailoski A.; Bowden P.; et al. OxLDL receptor chromatography from live human U937 cells identifies SYK(L) that regulates phagocytosis of oxLDL. Anal. Biochem. 2016, 513, 7–20. 10.1016/j.ab.2016.07.021. 27510553

[ref59] Dufresne J.; Bowden P.; Thavarajah T.; et al. The plasma peptides of ovarian cancer. Clin. Proteomics 2018, 15, 41 10.1186/s12014-018-9215-z. 30598658 PMC6302491

[ref60] Dufresne J.; Bowden P.; Thavarajah T.; et al. The plasma peptides of breast versus ovarian cancer. Clin. Proteomics 2019, 16, 43 10.1186/s12014-019-9262-0. 31889940 PMC6927194

[ref61] Thavarajah T.; dos Santos C. C.; Slutsky A. S.; et al. The plasma peptides of sepsis. Clin. Proteomics 2020, 17, 26 10.1186/s12014-020-09288-5. 32636717 PMC7331219

[ref62] Florentinus-Mefailoski A.; Bowden P.; Scheltens P.; et al. The plasma peptides of Alzheimer’s disease. Clin. Proteomics 2021, 18 (1), 17 10.1186/s12014-021-09320-2. 34182925 PMC8240224

[ref63] Leite J. F.; Dougherty D. A.; Lester H. A.; et al. Investigation of apparent mass deviations in electrospray ionization tandem mass spectrometry of a benzophenone-labeled peptide. Rapid Commun. Mass Spectrom. 2003, 17 (15), 1677–1684. 10.1002/rcm.1103. 12872270

[ref64] Putnam F. The plasma Proteins: Structure Function, and Genetic Control, 2nd ed.; Academic Press: New York, 1975.

[ref65] Marshall J.; Bowden P.; Schmit J. C.; et al. Creation of a federated database of blood proteins: a powerful new tool for finding and characterizing biomarkers in serum. Clin. Proteomics 2014, 11 (1), 3 10.1186/1559-0275-11-3. 24476026 PMC4015845

[ref66] Leinonen R.; Diez F. G.; Binns D.; et al. UniProt archive. Bioinformatics 2004, 20 (17), 3236–3237. 10.1093/bioinformatics/bth191. 15044231

[ref67] Craig R.; Cortens J. P.; Beavis R. C. Open source system for analyzing, validating, and storing protein identification data. J. Proteome Res. 2004, 3 (6), 1234–1242. 10.1021/pr049882h. 15595733

[ref68] Fenyö D.; Beavis R. C. A method for assessing the statistical significance of mass spectrometry-based protein identifications using general scoring schemes. Anal. Chem. 2003, 75 (4), 768–774. 10.1021/ac0258709. 12622365

[ref69] Craig R.; Beavis R. C. A method for reducing the time required to match protein sequences with tandem mass spectra. Rapid Commun. Mass Spectrom. 2003, 17 (20), 2310–2316. 10.1002/rcm.1198. 14558131

