# Supplementary material for: Comparison of the Human Plasma Peptides from the Fit of Fragmentation Spectra versus Accurate Monoisotopic Precursor Mass
Source: ACS Omega. 2025 Mar 10;10(11):10796–811. doi: 10.1021/acsomega.4c06211 (PMC11947786; doi:10.1021/acsomega.4c06211)
Supplement: Supplementary file 1 — ao4c06211_si_001.pdf [file ao4c06211_si_001.pdf]

Comparison of the human plasma peptides from the fit of fragmentation spectra versus accurate monoisotopic precursor mass

Zhuo Zhen Chen, Jaimie Dufresne, Peter Bowden and John G Marshall*

Research Analytical Biochemistry Laboratory, Department of Chemistry and Biology,

Faculty of Science, Toronto Metropolitan University, 350 Victoria Street, Toronto,

Ontario, Canada, M5B 2K3

***Corresponding author**

SUPPORTING INFORMATION

The characteristics of the common APOA1 peptide, LLDNWDSVTSTFSK from the orbital ion trap

The characteristics of the common APOA1 peptides from the LIT instrument versus the OIT instrument from the fit of MS/MS spectra

The false positive peptide p-value and peptide log p-value distribution of all random MS/MS spectra and those of Albumin (ALB) and Titan (TTN)

The peptide p-value and peptide log p-value distribution from uncorrected OIT MS/MS spectra ± 3 Da versus those of Albumin (ALB) and Titan (TTN)

The peptide p-value and gene symbol p-value distribution and log(n) distribution from all OIT MS/MS spectra ± 3 Da after noise and random MS/MS correction versus those of the accurate monoisotopic mass only and the true positive plasma proteins

Supplemental Table 1. The characteristics of the common APOA1 peptide, LLDNWDSVTSTFSK from the orbital ion trap. The Note the variation in MH and delta mass have little effect on the significance of the p-value from the fit of MS/MS spectra from -3 to +5 Da.

Precursor MZ	Precursor Intensity	Charge State	MH	Delta Mass	p-value	Missed Cleavages
806.1536255	211361795	2	1613.768775	-2.4694	5.3E-06	0
821.6781006	80091713.05	2	1644.774575	-2.4262	3.8E-06	0
805.6893921	2631433995	2	1612.784775	-2.4138	1.2E-08	0
813.8856201	16469339.84	2	1628.779775	-2.0164	4.7E-05	0
805.8931274	32768775.69	2	1612.784775	-2.0064	2.0E-08	0
821.8882446	24977932.84	2	1644.774575	-2.006	7.9E-05	0
806.0875854	104569332.9	2	1612.784775	-1.6174	2.9E-05	0
806.7283936	208147314	2	1613.768775	-1.3198	5.1E-05	0
806.3770142	11708664.98	2	1612.784775	-1.0386	0.0026	0
814.3811646	93160501.5	2	1628.779775	-1.0252	0.00025	0
537.9311523	6079016.715	3	1612.78415	-1.0062	0.0043	0
806.8938599	666915.4519	2	1613.768775	-0.9888	2.6E-06	0
806.5821533	1553014.148	2	1612.784775	-0.6282	0.00062	0
822.6751099	33535083.98	2	1644.774575	-0.4322	0.016	0
806.7574463	107710566.3	2	1612.784775	-0.2778	1.7E-06	0
814.7944336	50744048.06	2	1628.779775	-0.1988	1.7E-05	0
822.8883667	867326984.2	2	1644.774575	-0.0056	0.00019	0
822.8891602	78639230.98	2	1644.774575	-0.004	5.1E-05	0
814.8918457	68215226.79	2	1628.779775	-0.004	8.0E-05	0
814.8920898	91034840.28	2	1628.779775	-0.0034	4.1E-06	0
822.8895874	78539201.38	2	1644.774575	-0.0032	1.4E-06	0
806.8953857	126034392.8	2	1612.784775	-0.0018	1.4E-05	0
806.8959961	5438936.392	2	1612.784775	-0.0006	0.0022	0
814.8935547	31591786.39	2	1628.779775	-0.0004	0.0042	0
806.8963623	764428.8319	2	1612.784775	0.0002	5.4E-06	0
807.3966064	82426993.63	2	1613.768775	0.0166	0.0012	0
807.057373	1584978.929	2	1612.784775	0.3222	0.021	0
807.1276855	35738532.16	2	1612.784775	0.4628	0.005	0
807.630188	19028779.3	2	1613.768775	0.4838	0.00072	0
807.37146	152807076.4	2	1612.784775	0.9504	6.0E-08	0
823.390625	36981331.25	2	1644.774575	0.9988	8.6E-05	0
815.3933716	162040017.3	2	1628.779775	0.9992	0.005	0
807.3961792	156431739.5	2	1612.784775	0.9998	2.6E-05	0
823.3922729	38452886.5	2	1644.774575	1.0022	0.0055	0
823.3944702	277778126.8	2	1644.774575	1.0066	7.7E-05	0
815.4804688	62101431.25	2	1628.779775	1.1734	1.3E-07	0
807.8904419	157146625	2	1612.784775	1.9882	0.0011	0

Supplemental Table 2. The characteristics of the common APOA1 peptides from the LIT instrument versus the OIT instrument from the fit of MS/MS spectra. The Note the choice of instrument has little effect on the peptide p-value from the fit of MS/MS spectra from -3 to +5 Da. Bold OIT peptides are shared by both instruments. The results from a equal number of total peptides are shown.

**LIT_XTANDEM_APOA1**	**Mean p value**	**N**		**OIT X!TANDEM_APOA1**	**Mean p value**	**N**
LLDNWDSVTSTFSK	0.004120831	107		**LLDNWDSVTSTFSK**	0.001904365	37
WQEEMELYR	0.010842911	97		**WQEEMELYR**	0.020378464	28
DSGRDYVSQFEGSALGK	0.004105448	81		**QGLLPVLESFK**	0.004097411	27
LREQLGPVTQEFWDNLEK	0.005374161	77		**QKVEPLRAELQEGAR**	0.01665	23
VSFLSALEEYTK	0.001421101	73		**VQPYLDDFQKK**	0.025610476	21
DYVSQFEGSALGK	0.003758855	69		**LSPLGEEMR**	0.008485	18
VKDLATVYYDVLK	0.008006512	68		**DYVSQFEGSALGK**	0.009627508	17
KWQEEMELYR	0.011441485	59		**LREQLGPVTQEFWDNLEK**	0.004639911	16
QKVEPLRAELQEGAR	0.013403963	59		**KWQEEMELYR**	0.012156667	15
AKPALEDLR	0.019280702	57		**LEALKENGGAR**	0.015456667	15
QGLLPVLESFK	0.034863017	53		VKDLATVYYDVLK	0.012916602	15
ATEHLSTLSEK	0.008268677	51		**ATEHLSTLSEK**	0.006055	14
EQLGPVTQEFWDNLEK	0.005383472	48		**DSGRDYVSQFEGSALGK**	0.004822253	14
THLAPYSDELR	0.009182429	48		**LREQLGPVTQEFWDNLEKETEGLRQEMSK**	0.004959538	13
LEALKENGGAR	0.008368589	34		**QKLHELQEK**	0.002196154	13
DLATVYYDVLK	0.007708096	29		**AKPALEDLRQGLLPVLESFK**	0.004090195	12
QKLHELQEK	0.017919379	29		**THLAPYSDELR**	0.037205	12
VQPYLDDFQKK	0.0367708	25		**VQPYLDDFQK**	0.018916667	12
VEPLRAELQEGAR	0.00033245	20		**AKPALEDLR**	0.025699091	11
DLATVYYDVLKDSGRDYVSQFEGSALGK	0.011903375	18		**AKVQPYLDDFQKK**	0.009284362	11
LREQLGPVTQEFWDNLEKETEGLR	0.007157416	17		VQPYLDDFQKKWQEEMELYR	0.003201109	11
LSPLGEEMR	0.036417638	16		QGLLPVLESFKVSFLSALEEYTK	0.009782105	10
QKVEPLR	0.0551875	16		**QSPWDRVKDLATVYYDVLK**	0.0013397	10
THLAPYSDELRQR	0.042066772	15		RHFWQQDEPPQSPWDR	0.002461	10
DLATVYYDVLKDSGR	0.001339952	13		VKDLATVYYDVLKDSGRDYVSQFEGSALGK	0.004569855	10
VQPYLDDFQK	0.044815385	13		VSFLSALEEYTK	0.000601765	10
AHVDALR	0.054666667	12		**DLATVYYDVLK**	0.001203333	9
VKDLATVYYDVLKDSGRDYVSQFEGSALGK	9.18361E-07	12		**LAARLEALKENGGAR**	0.017967769	9
EQLGPVTQEFWDNLEKETEGLRQEMSK	0.011250891	11		VKDLATVYYDVLKDSGR	0.005278975	9
SALEEYTK	0.002115	11		LPDGLKLLDNWDSVTSTFSK	0.001113184	8
QEMSKDLEEVK	0.023767018	10		PLRAELQEGAR	0.000605816	8
VSFLSALEEYTKK	0.010224818	10		PYLDDFQKK	0.001017275	8

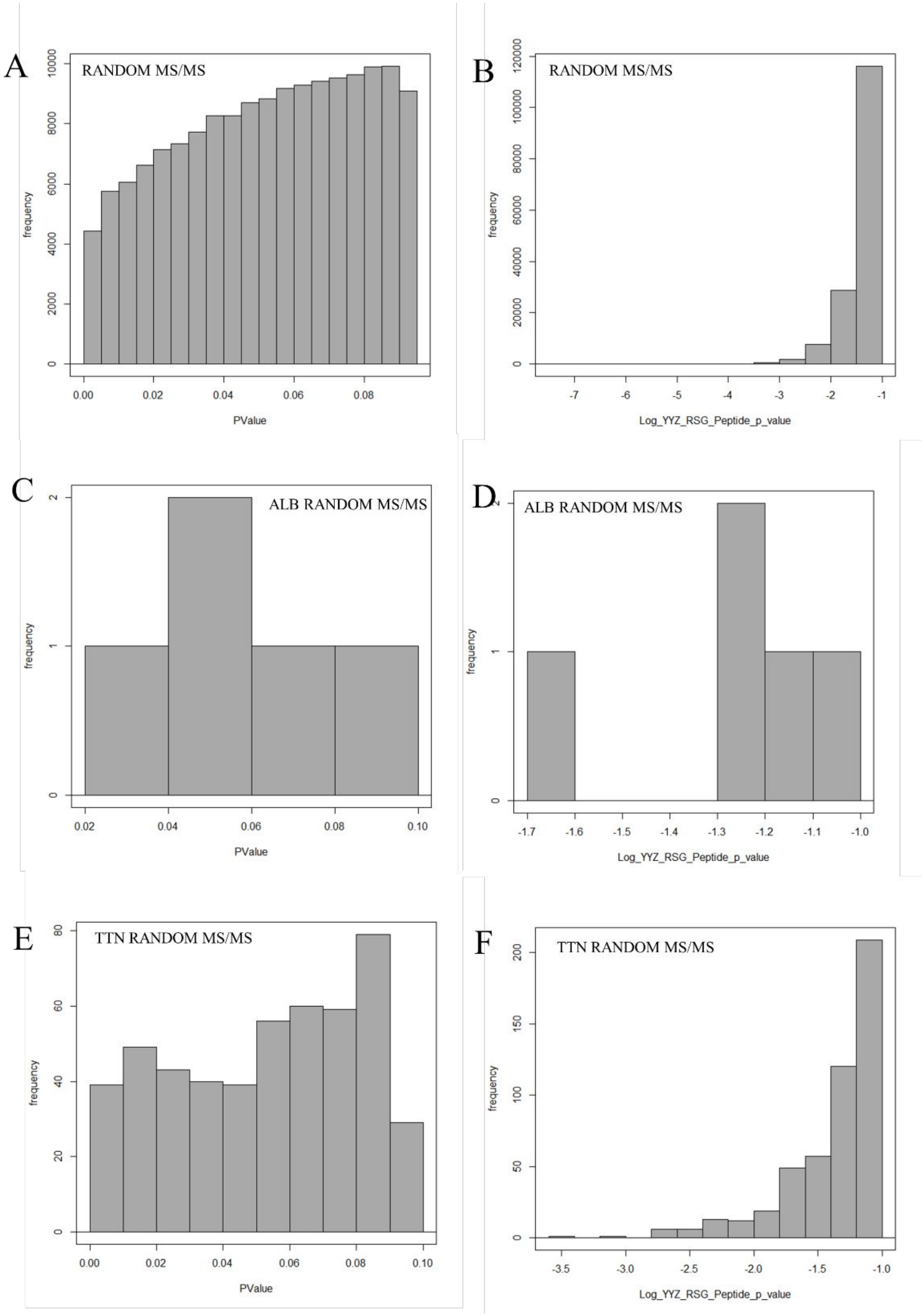

Supplemental Figure 1. The false positive peptide p-value and peptide log p-value distribution of all random MS/MS spectra and those of Albumin (ALB) and Titan (TTN). Panels: A&B the false positive distribution of 3 million random MS/MS spectra; C&D, the false positive distribution of Albumin (ALB); E&F, the false positive distribution of Titin (TTN). Note ALB has a low false positive frequency while TTN has a high false positive frequency. However, the false positive (Random MS/MS) p-value distribution of ALB and TTN are very similar.

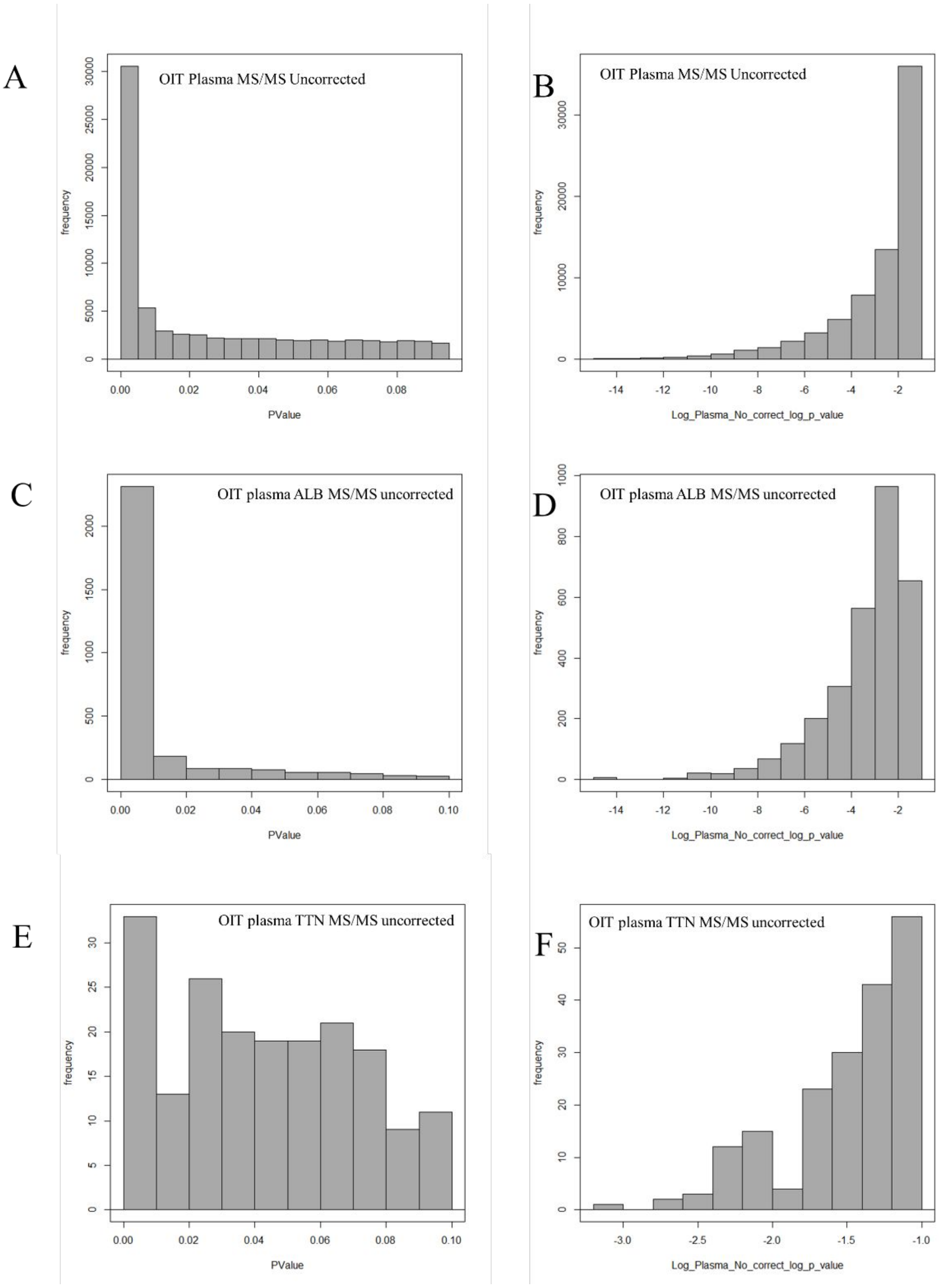

Supplemental Figure 2. The peptide p-value and peptide log p-value distribution from uncorrected OIT MS/MS spectra ± 3 Da versus those of Albumin (ALB) and Titan (TTN). Panels: A&B the uncorrected distribution of all MS/MS spectra ± 3 Da; C&D, the uncorrected distribution of Albumin (ALB); E&F, the uncorrected distribution of Titin (TTN). Note ALB has a high uncorrected observation frequency while TTN has a low uncorrected observation frequency.

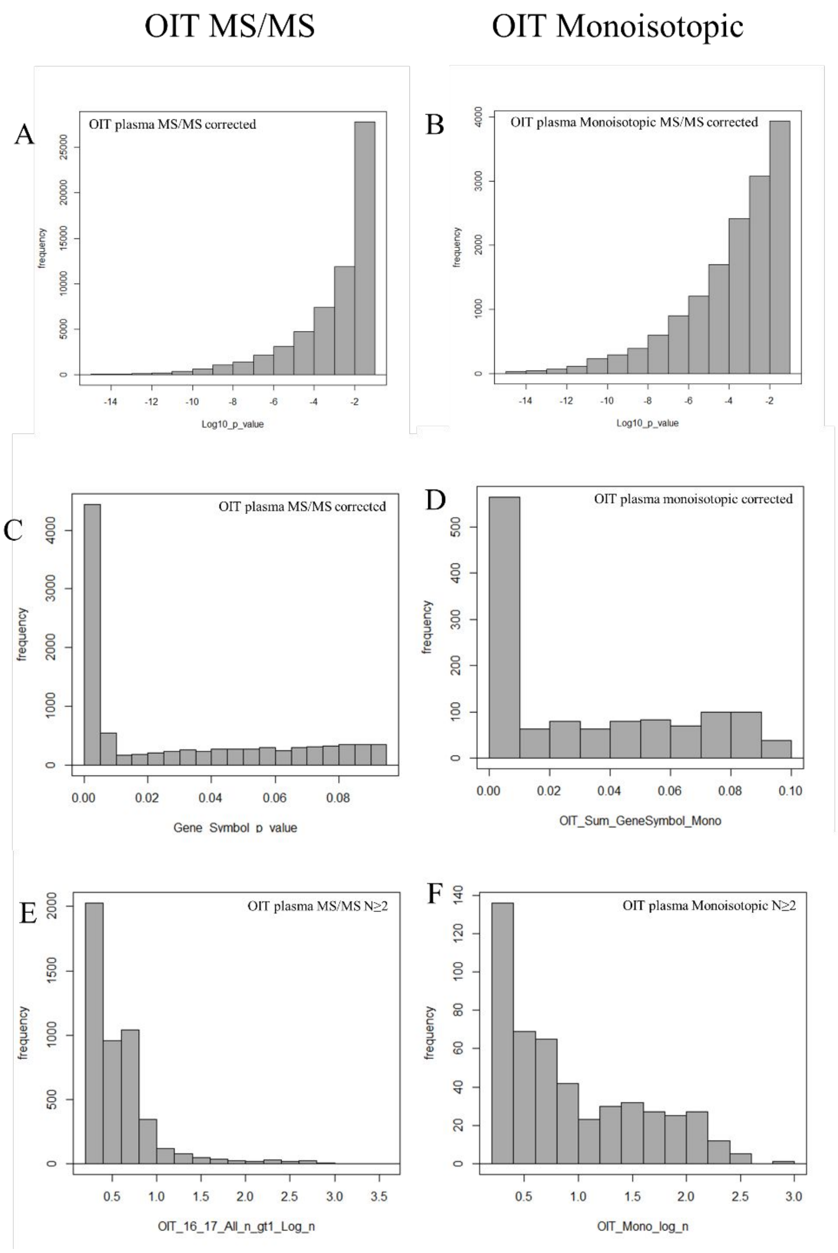

Supplemental Figure 3. The peptide p-value and gene symbol p-value distribution and log(n) distribution from all OIT MS/MS spectra ± 3 Da after noise and random MS/MS correction versus those of the accurate monoisotopic mass only and the true positive plasms proteins where $N \geq 2$ $q<0.01$. Panels: A&B, peptides and log peptide distribution; C&D, protein gene symbols; E&F, protein gene symbols p-value distribution where ($N \geq 2$) and FDR less than 1% ($q \leq 0.01$). Note that true positive identifications are derived from the single best fit per spectra after correction against noise and random MS/MS spectra before computing the protein p-value in X!TANDEM and selecting proteins with an FDR q value $N \geq 2$ and $q \leq 0.01$ true positive identification. Note that the monoisotopic OIT result ±0.1 Da provides few true positive proteins while the OIT ±3 Da provide about 10-fold more protein gene symbols with significant p-values.
